# Epigenetic Dysregulation in Neurodegeneration: The Role of Histone Deacetylases and Emerging Inhibitor Strategies

**DOI:** 10.3390/biom16010103

**Published:** 2026-01-07

**Authors:** Yogesh Pawar, Aleksandra Kopranovic, Ramaa C S, Franz-Josef Meyer-Almes

**Affiliations:** 1 Department of Pharmaceutical Chemistry, Bharati Vidyapeeth’s College of Pharmacy, CBD Belapur, Navi Mumbai 400614, India; 2 Department of Chemical Engineering and Biotechnology, University of Applied Science, 64290 Darmstadt, Germany; 3 European University of Technology, European Union, 64290 Darmstadt, Germany

**Keywords:** histone deacetylase inhibitors (HDACis), neurodegenerative diseases, Alzheimer’s disease (AD), Parkinson’s disease (PD), Huntington’s disease (HD), glioblastoma, s-traizine, blood–brain barrier permeability, multi-target-directed ligands (MTDLs)

## Abstract

Neurodegenerative diseases such as Alzheimer’s disease (AD), Parkinson’s disease (PD), and Huntington’s disease (HD) are characterized by complex pathologies with progressive neurodegeneration, protein misfolding, oxidative stress, and persistent inflammation. Recent findings indicate the pivotal involvement of epigenetic disruption, particularly aberrant histone deacetylase (HDAC) activity, in disease initiation and progression. In the current review, we systematically discuss the mechanistic function of HDACs across all classes (I, IIa, IIb, III, and IV) in neurodegenerative disease mechanisms, such as their involvement in the modulation of gene expression, mitochondrial function, proteostasis, and neuronal survival. We discuss the therapeutic potential, as well as limitations, of HDAC inhibitors (HDACis), such as pan-inhibitors and isoenzyme-selective inhibitors, and new multi-target-directed ligands with HDAC inhibition combined with acetylcholinesterase modulation, PDE modulation, MAO-B inhibition, or NMDAR modulation. Particular emphasis is placed on the development of HDAC6-selective inhibitors with enhanced brain permeability and reduced toxicity, which have shown promising preclinical efficacy in ameliorating hallmark pathologies of AD, PD, and HD. In addition, s-triazine-based scaffolds have recently emerged as promising chemotypes in HDAC inhibitor design, offering favorable pharmacokinetic profiles, metabolic stability, and the potential for dual-target modulation relevant to neurodegeneration. The review also explores the future of HDAC-targeted therapies, including PROTAC degraders, dual-inhibitor scaffolds, and sustainable, BBB-penetrant molecules. Collectively, this review underscores the importance of HDAC modulation as a multifaceted strategy in the treatment of neurodegenerative diseases and highlights the need for continued innovation in epigenetic drug design.

## 1. Introduction

Neurological disorders are multidimensional and multifaceted complex diseases that impact millions of individuals worldwide by disrupting the fundamental mechanisms that regulate thoughts, movements, and emotions [1]. The origins are diverse; some result from structural damage, disrupted brain connectivity, or signaling dysfunctions, making them particularly difficult to diagnose and treat [2]. Building on this complexity, the burden of deaths and disability caused by neurological disorders is increasingly recognized as a global public health challenge, the global burden of neurological disorders is increasingly acknowledged as a major public health threat. Alzheimer’s disease and other dementias are some of the greatest causes of disability for elderly people, with rising numbers mainly due to an aging population and longer life spans. According to Global Burden of Disease (GBD) 2021 estimates, around 36.3 million DALYs were attributed to dementia globally. Parkinson’s disease (PD) is the fastest-growing neurological disorder, and it was responsible for 7.4 million DALYs in 2021, reflecting a substantial increase over previous decades, with an estimated global burden of 25.2 million by the year 2050 [3,4,5].

Together, these trends highlight the rising global burden of neurodegenerative diseases and the need for effective therapies and stronger healthcare systems. Although less prevalent, disorders such as glioblastoma and Huntington’s disease impose significant clinical and societal impacts, while neurological diseases broadly stem from brain network dysfunctions affecting the central and peripheral nervous systems and causing diverse cognitive and sensory symptoms [6].

The degeneration is primarily driven by the pathological accumulation of misfolded proteins and other toxic molecules [7,8]. These pathological changes lead to a progressive decline in cognitive impairments, motor deficits, and emotional disturbances, impacting an individual’s quality of life [9]. Early detection and treatment are fundamental for controlling neurodegenerative diseases. In vivo detection of disease-specific molecular abnormalities is possible using positron emission tomography (PET), which helps in accurate diagnosis, staging, and treatment response measurement. When combined with atomic force microscopy (AFM) offers label-free nanoscale probing of pathological protein aggregation and nanomechanical changes, a multimodal approach further extends mechanistic insight and biomarker discovery to a variety of neurodegenerative diseases such as Alzheimer’s, Parkinson’s, and Huntington’s. Table 1 summarizes complementary roles of PET and AFM imaging in probing molecular, functional, and biophysical changes that characterize Neurodegenerative disorders [10,11].

These protein aggregates trigger a cascade of pathogenic cellular mechanisms, including proteotoxic stress, increased oxidative damage, activation of cell death pathways, and sustained neuroinflammatory responses, all contributing to the progressive degeneration of neural tissue [12,13]. Given their widespread and serious impact, understanding the underlying mechanisms of neurological disorders is vital for developing targeted treatments aimed at restoring brain function and improving patient outcomes [14,15,16]. Recently, there has been a growing focus on epigenetic mechanisms, which significantly impact the development and progression of these conditions.

**Figure 1:**
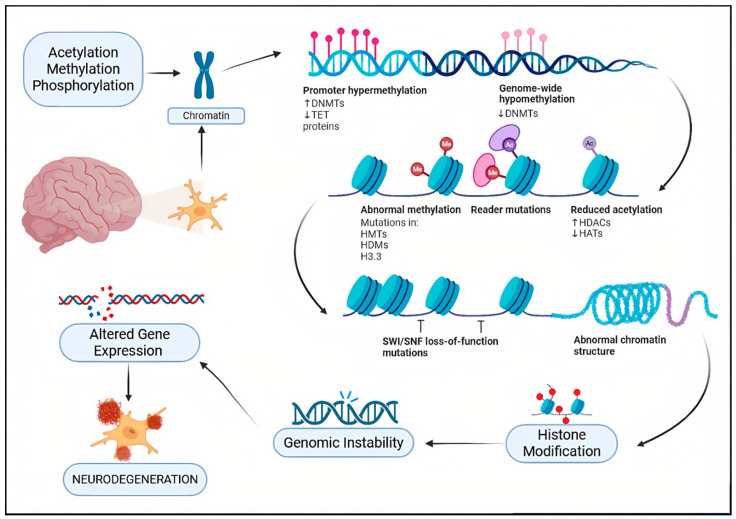
Epigenetic mechanisms regulating chromatin structure and gene expression.

Epigenetics refers to heritable change in gene activity and expression that do not alter the underlying DNA sequence. The idea was first presented by Conrad Waddington in 1940, which describes how environmental influences might affect gene expression without altering the DNA itself in ways that are inherited by subsequent generations [17,18]. These modifications can either activate or silence genes and are influenced by developmental, environmental, and lifestyle factors [19]. At the molecular level, DNA is compacted into chromatin by wrapping around histones. Each nucleosome, the basic chromatin unit, contains DNA wound around core histones H2A, H2B, H3, and H4, with H1 linking nucleosomes to maintain higher-order structure [20,21,22]. Epigenetic regulation of gene expression is primarily governed by DNA methylation, histone modifications, and RNA-mediated mechanisms. These alterations are rarely inherited and are typically reversible. recently, a lot of treatments focus on the epigenetic regulation of gene expression, particularly post-translational modifications of proteins [23]. The histone deacetylase (HDAC) enzyme family has received significant attention among these. Since our focus is primarily on histone modifications, it is important to outline the diverse post-translational changes histones can undergo. These include acylation, which comprises acetylation, benzoylation, butyrylation, crotonylation, glutarylation, and lactylation, as well as modifications such as ADP-ribosylation, dopaminylation, glycosylation, methylation, phosphorylation, serotonylation, sumoylation, and ubiquitination [24,25]. Together, these modifications create a regulatory code that governs gene expression and presents a promising avenue for targeted treatments. By eliminating acetyl and other acyl groups from lysine residues on histone and non-histone proteins, histone deacetylases (HDACs) are important epigenetic enzymes that affect transcription, cell differentiation, DNA replication, and the cell cycle [26]. Functioning in concert with histone acetyltransferases (HATs), HDACs maintain the delicate balance of lysine acetylation/acylation within cells [27]. Disruption of this balance has been strongly associated with the development of cancers, autoimmune conditions, and neurological disorders, underscoring the therapeutic potential of targeting these enzymes [28] (Figure 1).

Building on this, growing evidence indicates that in neurodegenerative disorders such as Parkinson’s disease (PD), Alzheimer’s disease (AD), Huntington’s disease (HD), and Amyotrophic lateral sclerosis (ALS), by regulating chromatin dynamics and gene expression that affect neuronal survival, synaptic plasticity, and inflammation [29,30]. In Parkinson’s disease, degeneration of dopaminergic neurons in the substantia nigra causes motor and cognitive symptoms and is associated with α-synuclein-containing Lewy bodies [31,32]. Dysregulated HDAC activity promotes neuronal vulnerability through impaired acetylation, mitochondrial dysfunction, and oxidative stress, while inhibition of HDAC isoenzymes such as HDAC4 and HDAC6 may protect neurons by enhancing autophagy and cellular homeostasis [33,34]. Alzheimer’s disease is characterized by extracellular amyloid-β plaques and intracellular tau tangles, arising from multifactorial genetic and environmental factors [35,36]. The etiology of the disease is multifactorial, and genetic vulnerabilities in combination with environmental and lifestyle factors causes mutations in APP, PSEN1/2, and APOE ε4 alleles, HDAC2 and HDAC6 suppress genes involved in synaptic plasticity and memory, and their inhibition has been shown to restore cognitive function, reduce tau pathology, and improve mitochondrial activity in AD models [37,38,39]. Huntington’s disease is a genetic neurodegenerative disorder caused by the expansion of the CAG trinucleotide repeat in the Huntingtin gene (HTT) that produces a mutant huntingtin protein (mHTT) [40]. The disruption of cellular processes like transcription, mitochondrial dynamics, and protein degradation is caused by the mutation of proteins. Aberrant activity of class IIa HDACs, including HDAC4 and HDAC5, worsens neuronal toxicity, while selective HDAC inhibition restores transcriptional balance, reduces mutant protein toxicity, and improves motor and cognitive outcomes in preclinical models [41,42,43]. Amyotrophic lateral sclerosis (ALS) involves progressive degeneration of motor neurons, leading to muscle weakness, paralysis, and respiratory failure, and is marked by intracellular protein aggregates such as TDP-43. Dysregulated HDAC activity promotes motor neuron vulnerability through impaired acetylation, mitochondrial dysfunction, and neuroinflammation, making selective inhibition of HDAC2, HDAC4, and HDAC6 a promising therapeutic strategy [44].

Given the multifaceted nature of these illnesses, oxidative stress, mitochondrial damage, failure in proteostasis, and neuroinflammation, a multi-target therapeutic approach that includes modulation of HDAC activity is highly promising [45]. In contrast to single-target approaches, which in general are not sufficient in addressing the multifaceted pathology of neurodegenerative illnesses, HDAC inhibitors confer the advantage of modulating several disease pathways concurrently [46,47]. Such widespread activity renders HDACs excellent targets for the discovery of next-generation neuroprotectants. Despite promising preclinical results, HDAC inhibitors (HDACis) have not yet translated into effective clinical treatments for neurological disorders. Drugs such as vorinostat and panobinostat exhibit poor blood–brain barrier (BBB) penetration and, in Phase I and Phase II clinical trials for Alzheimer’s and glioblastoma, have induced dose-limiting toxicities, including hematological and gastrointestinal side effects [48,49]. Givinostat, though promising in inflammatory conditions like Duchenne muscular dystrophy, lacks sufficient evidence in classical neurodegenerative diseases. Valproic acid, while widely used for epilepsy and mood disorders, has shown only modest benefits in diseases such as Alzheimer’s disease and ALS in Phase II and Phase III clinical trials, likely due to its broad, non-selective mechanism of action [50,51]. A major challenge lies in the lack of isoenzyme selectivity of most approved HDACis, which are pan-inhibitors and therefore have an increased risk of off-target effects [52]. Additionally, inadequate CNS bioavailability further limits their utility in treating brain-specific disorders [53]. To overcome these issues, future approaches should prioritize isoenzyme-selective HDAC inhibitors with enhanced CNS penetration, supported by advanced delivery systems such as prodrugs or nanoparticles. Combination therapies with neuroprotective or gene-modulating agents may further improve efficacy while reducing toxicity [14]. Timing is equally crucial; HDACis may offer greater benefit when administered during early stages of neuroinflammation or epigenetic dysregulation [54].

This review explores the role of epigenetic regulation in neurological disorders, particularly focusing on histone deacetylases (HDACs) and the impact of nitrogen-containing small-molecule modulators thereof. It highlights the multifaceted involvement of HDACs in the progression of neurodegenerative diseases such as AD, PD, HD, and glioblastoma and evaluates the therapeutic promise of HDAC inhibitors. By uncovering the molecular mechanisms linked to HDAC activity, this work aims to offer a broad perspective on how modulating these enzymes could support the development of more effective, multi-targeted strategies in neuroepigenetic therapy.

## 2. Histone Deacetylases: Functional Roles and Therapeutic Potential

Histone deacetylases (HDACs) are epigenetic regulators that influence chromatin organization and gene expression by modulating lysine acetylation in opposition to histone acetyltransferases [55,56]. Histone acetyltransferases activate transcription by relaxing chromatin, while HDACs repress gene expression by reversing this modification and regulating key non-histone proteins involved in cellular signaling and DNA repair, highlighting their relevance in neurological disorders. HDACs remove acetyl and other acyl groups from lysine residues on histone and non-histone proteins across nuclear, cytosolic, and mitochondrial compartments. Mammalian HDACs comprise 18 enzymes grouped into four classes: zinc-dependent classes I, II, and IV, and NAD^+^-dependent class III sirtuins. Class I HDACs are ubiquitously expressed and essential for neural development and synaptic function, while class II HDACs mainly regulate non-histone substrates involved in neuronal signaling and transport. Class IV HDAC11 is implicated in immune–neuronal regulation, and class III sirtuins play key roles in neuronal survival, aging, and neurodegeneration [57,58].

HDAC inhibitors share a common pharmacophore consisting of a cap group, connecting unit, linker, and zinc-binding group. The cap mediates surface recognition, the connecting unit optimizes molecular orientation, and the linker extends into the hydrophobic channel to reach the catalytic site [59]. The zinc-binding group (ZBG) is essential for HDAC inhibition, as it chelates the catalytic zinc ion and largely determines inhibitor potency and selectivity. Targeting the ZBG is therefore central to designing effective and safer HDAC inhibitors. Dysregulated HDAC activity is implicated in neurological and neurodegenerative disorders, including Alzheimer’s disease, where HDAC2 overexpression drives synaptic loss and memory impairment [60].

Parkinson’s disease, where HDAC6-mediated protein misfolding and impaired autophagy exacerbate dopaminergic neuronal death; Huntington’s disease, characterized by transcriptional dysregulation and toxic protein aggregation responsive to HDAC inhibition; and glioblastoma, where HDACs modulate tumor cell proliferation, immune evasion, and therapeutic resistance [61,62].

As such, HDAC inhibitors (HDACis) have emerged as a promising class of epigenetic therapeutics aimed at reactivating silenced neuroprotective genes, attenuating neuroinflammation, restoring synaptic plasticity, and promoting neuronal survival. These inhibitors, which include pan-HDACis (e.g., vorinostat, panobinostat), class-selective inhibitors (e.g., entinostat for Class I, tubacin for HDAC6), and brain-penetrant agents (e.g., valproic acid), have demonstrated beneficial effects in preclinical models, though clinical translation faces challenges such as off-target toxicity, poor blood–brain barrier permeability, and isoenzyme non-specificity [43,63]. Given the limitations associated with broad-spectrum HDAC inhibition, increasing emphasis has been placed on the development of isoenzyme-selective HDAC inhibitors. A comparative overview of pan-HDAC and isoenzyme-selective HDAC inhibitors is presented in Table 2.

Ongoing research is focused on refining HDACi selectivity, optimizing CNS delivery systems, and developing combination therapies that enhance neurodegenerative outcomes while minimizing adverse effects, making HDAC modulation a key frontier in treating neurological disorders with epigenetic underpinnings (Table 3).

## 3. HDAC Inhibitors in the Treatment of Neurodegenerative Disorders

### 3.1. Alzheimer’s Disease

Alzheimer’s disease (AD) is a leading cause of dementia, progressively impairing memory, cognition, and higher-order functions, ultimately causing severe disability and death [79]. First identified by Alois Alzheimer in 1906 [81], AD is classified by onset: rare early-onset AD (EOAD) results from mutations in APP, PSEN1, or PSEN2, while the more common late-onset AD (LOAD) occurs sporadically in older adults, with epigenetic factors increasingly recognized as key contributors to disease progression [82].

In Alzheimer’s disease, Nuclear histone deacetylases (HDACs), particularly class I enzymes such as HDAC1 and HDAC2, remove acetyl groups from histones, leading to chromatin condensation and transcriptional repression of genes essential for neuronal function, repair, and synaptic plasticity [83,84]. Epigenetic mechanisms, particularly histone acetylation regulated by HATs and HDACs, play a crucial role in gene expression and are disrupted in neurodegeneration [85,86]. HDAC-dependent repression reduces neprilysin (NEP), a principal Aβ-degrading enzyme, thereby promoting Aβ oligomers and plaque pathology in AD; α-synuclein oligomers also accumulate in AD/DLB via partly distinct proteolytic pathways [87,88].

HDAC2, a class I histone deacetylase, plays a key role in Alzheimer’s disease by repressing genes critical for synaptic plasticity, learning, and memory, such as BDNF, Arc, EGR1, and CREB-regulated pathways. Its overexpression in the AD brain causes histone hypoacetylation, chromatin condensation, and transcriptional silencing, which strongly correlates with cognitive decline and dendritic spine loss. Beyond epigenetic effects, HDAC2 also modulates non-histone proteins, promoting amyloid-β accumulation, tau hyperphosphorylation, oxidative stress, neuroinflammation, mitochondrial dysfunction, and blood–brain barrier disruption through pathways including NF-κB, JAK/STAT, and MAPK. Pharmacological or genetic inhibition of HDAC2 restores histone acetylation, reactivates neuroprotective genes, and improves cognitive function in experimental models. These findings highlight HDAC2 as a disease-modifying therapeutic target and support the development of HDAC2-selective, brain-penetrant inhibitors and multi-target-directed ligands to overcome limitations of non-selective HDAC inhibitors and more effectively address AD pathology [89,90].

**Figure 2:**
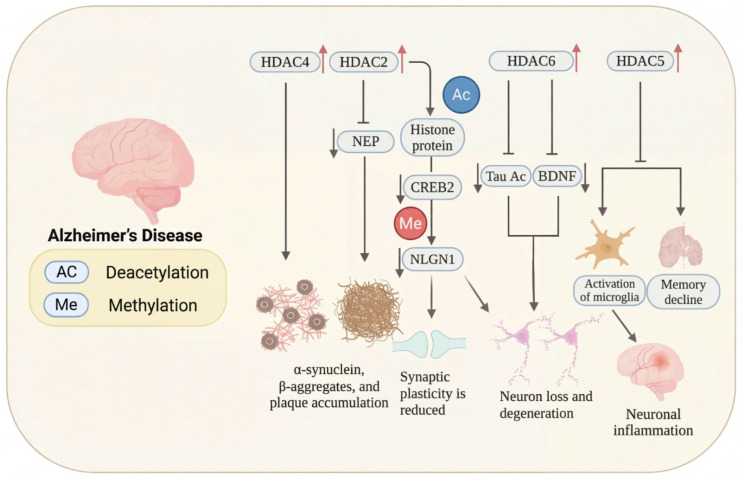
HDAC-driven epigenetic modulation in Alzheimer’s disease: impacts on protein aggregation, synaptic plasticity, and memory decline [58].

HDAC6 causes additional loss by inducing the accumulation of Tau protein, which creates neurofibrillary tangles within neurons, impairing cell function. It also reduces the levels of brain-derived neurotrophic factor (BDNF), an essential molecule to maintain neuron survival, growth, and differentiation. The reduction in BDNF also induces the activation of microglia, the brain’s immune cells. Upon hyperactivation, microglia trigger inflammatory processes, which enhance neuronal injury and neuronal loss [37,91]. HDAC5, though not specifically related to molecular mechanisms in this figure, plays a role in the overall outcome of memory loss, so it makes sense how several HDACs function together in cognitive dysfunction [92] (Figure 2).

#### 3.1.1. HDAC Inhibitors in Alzheimer’s Disease Therapy

**Figure 3:**
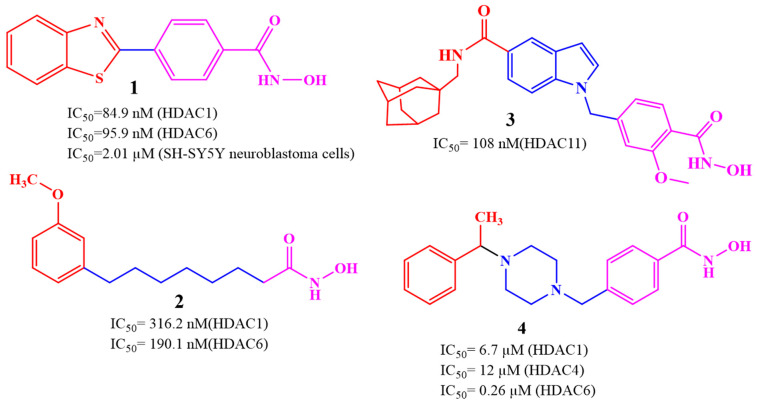
Structures of chemotherapeutic agents studied as HDAC inhibitors.

HDAC inhibitors are emerging as potential Alzheimer’s therapies. Choi et al. (2019) developed CNS-penetrant benzoheterocyclic HDAC inhibitors inspired by amyloid-β imaging agents [93]. Compound **1**, a benzothiazole derivative, showed potent antiproliferative activity against SH-SY5Y neuroblastoma cells (IC_50_ = 2.01 μM) and selectively inhibited HDAC1 (IC_50_ = 84.9 nM) and HDAC6 (IC_50_ = 95.9 nM) (Figure 3). It increased acetylated histone H3 and α-tubulin levels, indicating effects on chromatin remodeling and microtubule stability. PAMPA-BBB assays (Pe = 27.15) and in vivo mouse studies revealed a 29-fold higher brain concentration than SAHA. Molecular docking confirmed binding to HDAC1 and HDAC6 via zinc coordination and hydrophobic interactions, highlighting its strong potential for AD therapy. Romeiro et al. in 2019 introduced a novel and sustainable approach to developing histone deacetylase inhibitors (HDACis) for Alzheimer’s disease (AD) by deriving them from cashew nutshell liquid (CNSL), an agro-food waste product [94]. Inspired by the FDA-approved HDAC inhibitor vorinostat, the authors synthesized two hydroxamate-based compounds, designed to inhibit class I HDAC1 and class IIb HDAC6, both of which are implicated in AD pathophysiology. These compounds demonstrated effective HDAC inhibition (compound **2**: HDAC1 IC_50_ = 316.2 nM; HDAC6 IC_50_ = 190.1 nM) (Figure 3), showing a better safety profile in primary neuronal cells compared to vorinostat. Importantly, both compounds showed good blood–brain barrier permeability in PAMPA assays. The compounds also exhibited neuroprotective effects at the cellular level by increasing histone H3 acetylation and modulating microglial inflammatory responses. Notably, they reduced the proinflammatory marker iNOS, it also promoted expression of the anti-inflammatory MRC1 marker, indicating a potential shift toward a neuroprotective microglial phenotype. Although they did not rescue neurons under severe deprivation conditions, compound **2** was non-neurotoxic and better tolerated than vorinostat, highlighting CNSL-derived HDAC inhibitors as promising, low-cost, and sustainable leads for AD therapy.

Along these lines, compound **3** is a brain-penetrant, selective inhibitor of HDAC11 with favorable pharmacokinetic properties and central nervous system availability, developed by Bai et al. (2025) [81]. HDAC11 has been shown to colocalize with amyloid plaques and neuroinflammatory markers, suggesting a contributory role in AD pathology. Derived from an indole-adamantane-based hydroxamic acid scaffold originally targeting HDAC6 and HDAC11, compound **3** (Figure 3) was optimized to exhibit strong potency (IC_50_ = 108 nM) and approximately 40-fold selectivity toward HDAC11 over other HDAC isoenzymes. In HEK293T cells, compound **3** increased fatty acylation of serine hydroxymethyltransferase 2 (SHMT2), a known HDAC11 substrate, without inducing cytotoxicity at concentrations up to 3.3 μM. Compound **3** showed favorable in vitro ADME properties, with good metabolic stability (t_1_/_2_ = 54.6 min in human liver microsomes, 133.8 min in mouse plasma) and no significant inhibition of CYP1A2, 2C19, or 2D6 at 10 μM, suggesting low drug–drug interaction potential. In mice, it displayed a 5.3 h oral half-life at 10 mg/kg, though with modest oral bioavailability (11.2%). Importantly, after intraperitoneal administration, 3 demonstrated effective brain penetration. Functionally, 3 reduces amyloid burden in AD mouse models, as demonstrated by immunohistochemistry (IHC), positron emission tomography (PET), and chemiluminescence imaging. It also improves cognitive performance, as evidenced by enhanced outcomes in the Y-maze, novel object recognition, and buried food tests [81]. Additionally, compound **3** mitigates neuroinflammation by promoting Aβ phagocytosis in BV2 microglial cells, decreasing cytokine levels of IL-5 and IL-10, and increasing expression of the chemokine KC/GRO. Overall, the study highlights HDAC11 as a novel and druggable target for Alzheimer’s disease and positions compound **3** as a promising lead compound for further therapeutic development.

Among the recently developed HDAC6 inhibitors with optimized CNS penetration, compound **4** represents a notable example of a hybrid-designed compound (Figure 3). Compound **4**, a benzylpiperazine-derived hydroxamic acid, was developed by Hashimoto et al. (2022) using a hybrid strategy that integrates HDAC6 inhibitory scaffolds with blood–brain barrier (BBB)-permeable features from H1-antihistamines like cetirizine for treating central nervous system (CNS) disorders, including Alzheimer’s disease [95]. Compound **4** demonstrated strong selectivity for HDAC6 (IC_50_ = 0.26 μM) over HDAC1 and HDAC4, along with a low risk of cardiotoxicity (hERG IC_50_ = 106 μM). It possessed favorable solubility, metabolic stability, and an excellent brain/plasma concentration ratio of 6.73, confirming efficient CNS uptake. In mouse models, compound **4** significantly increased acetylated α-tubulin levels in the brain without altering histone H3 acetylation, confirming cytoplasm-specific HDAC6 inhibition. Additionally, 4 produced antidepressant-like effects in behavioral tests, supporting its activity in the CNS.

**Figure 4:**
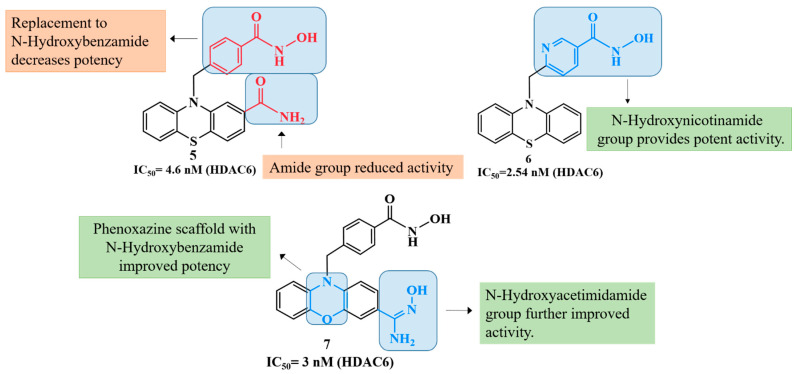
Evolution of scaffold modification showing the influence of hydroxamic acid derivatives on HDAC inhibition potency.

Building on similar rationales, Hsu et al. in their 2021 study designed and synthesized phenothiazine-based hydroxamic acid derivatives with, the aim of selectively inhibiting class II histone deacetylases (HDACs), particularly HDAC6 [96]. Phenothiazine was chosen for its unique non-planar structure and known neuroprotective and antioxidant properties. Among the synthesized compounds, compound **5** (Figure 4) showed potent HDAC6 inhibition (IC_50_ = 4.6 nM) with high selectivity over class I HDACs. Docking confirmed binding to the HDAC6 catalytic site via zinc coordination, hydrogen bonding, and π–π interactions. In SH-SY5Y cells, compound **5** increased acetylated α-tubulin and histone H3 levels without notable cytotoxicity, provided neuroprotection against H_2_O_2_-induced oxidative stress (cell viability > 70%), and promoted neurite outgrowth, supporting its potential as a selective, neuroprotective HDAC6 inhibitor for Alzheimer’s therapy.

Building upon the evidence of CNS permeability and therapeutic potential of compound **6**, subsequent studies have further advanced the field by developing structurally diverse and highly selective HDAC6 inhibitors, Wang et al. (2021) developed phenothiazine-based HDAC6 inhibitors, with compound **6** (Figure 4) showing exceptional potency (IC_50_ = 2.54 nM) and 290–3399-fold selectivity over other HDACs [97]. Docking confirmed zinc coordination and stabilizing π–π and hydrogen-bond interactions, similar to tubastatin A. In SH-SY5Y cells, 6 selectively increased α-tubulin acetylation, reduced tau phosphorylation at Ser396, chelated Cu^2+^, and inhibited Aβ_1–42_ aggregation (~40% ThT reduction), restoring cell viability to 82%. It also enhanced neurite outgrowth and upregulated neurogenesis markers (GAP43, N-myc, MAP-2), demonstrating CNS activity, neuroprotection, and multi-target therapeutic potential for Alzheimer’s disease.

Building on these advances, new structural frameworks have been explored to broaden the spectrum of HDAC inhibition, Chu et al. (2023), reports the synthesis and biological evaluation of C-4 substituted phenoxazine-bearing hydroxamic acids as potent and selective class II histone deacetylase (HDAC) inhibitors [98]. The lead compound **7** (Figure 4), demonstrated exceptional activity, inhibiting HDAC6 with an IC_50_ of 3 nM, along with potent inhibition of class IIa HDACs (HDAC4, 7, 9) and modest activity against class I isoenzymes. Molecular docking studies revealed that 7 interacts robustly with HDAC6 and HDAC7. Functionally, compound **7** promoted α-tubulin acetylation (a marker of HDAC6 inhibition) more effectively than SAHA and only modestly affected histone acetylation, confirming its selectivity toward HDAC6 over class I HDACs. In SH-SY5Y neuroblastoma cells, **7** showed low cytotoxicity and superior neuroprotective effects against H_2_O_2_-induced oxidative stress, restoring cell viability in a dose-dependent manner. These results position compound **7** as a highly selective, non-toxic, and neuroprotective HDAC inhibitor with significant potential for further development as a therapeutic agent in Alzheimer’s disease.

The structure–activity relationship (SAR) illustrated in the figure (Figure 4) demonstrates the impact of structural modifications on the biological potency of hydroxamic acid derivatives. Introduction of the N-hydroxynicotinamide moiety in compound **6**, resulted in significantly enhanced activity, highlighting its effective zinc-binding ability and favorable electronic characteristics. In contrast, replacement of this group with N-hydroxybenzamide led to a marked reduction in potency in compound **5**, indicating the importance of the pyridine nitrogen in maintaining optimal chelation and target interaction. Furthermore, incorporation of an additional amide group was found to further diminish activity, possibly due to steric hindrance or altered hydrogen bonding. Interestingly, substitution of the core scaffold with a phenoxazine ring restored and enhanced potency, particularly when combined with the N-hydroxybenzamide fragment in compound **7**, suggesting that the extended conjugation and heteroatom framework of phenoxazine contributed to improved binding affinity. Subsequent modification with the N-hydroxyacetamide group resulted in further enhancement of activity, confirming that careful optimization of both the zinc-binding domain and the aromatic scaffold is crucial for maximizing biological efficacy. While initially evaluated for depression, the compound’s brain-targeting ability, HDAC6 specificity, and functional effects on cytoplasmic acetylation align well with therapeutic goals in AD, particularly in modulating tau pathology and neuronal transport deficits.

#### 3.1.2. Dual-Target Inhibitors (HDAC + Secondary Target)

**Figure 5:**
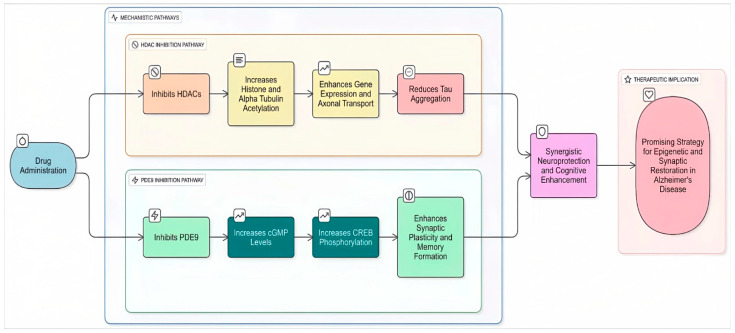
Synergistic neuroprotection and cognitive enhancement via dual HDAC and PDE9/PDE5 inhibition.

Phosphodiesterases (PDEs) are enzymes that regulate levels of key signaling molecules in the brain, and their dysfunction is increasingly recognized as playing a significant role in Alzheimer’s disease [99]. PDEs regulate cyclic AMP (cAMP) and cyclic GMP (cGMP), which are crucial for memory, synaptic plasticity, and neuronal survival. Inhibiting specific PDEs restores cAMP/cGMP signaling, activates CREB, and increases neuroprotective factors like BDNF [100]. This leads to improve memory, reduce neuroinflammation, and decrease amyloid-beta pathology in preclinical models, making PDEs promising therapeutic targets for AD and secondary targets in Dual-Target Inhibitors (Figure 5).

Sánchez Arias et al. (2017) developed dual HDAC/PDE5 inhibitors for Alzheimer’s disease using vardenafil- and tadalafil-based scaffolds [101]. Compound **8** (vardenafil-based) (Table 4) inhibited PDE5 (IC_50_ = 0.27 nM) and HDAC6 (IC_50_ = 110 nM), enhancing histone H3 and tubulin acetylation, CREB phosphorylation, and reducing APP processing and tau phosphorylation in SH-SY5Y and Tg2576 neurons, but showed moderate cytotoxicity (LC_50_ ≈ 6–9 µM), low brain permeability (logBB = −1.9), and poor solubility. Compound **9** (tadalafil-based) (Table 4) inhibited HDAC1 (IC_50_ = 68 nM) and HDAC6 (IC_50_ = 77 nM) with modest PDE5 activity, inducing moderate histone acetylation and CREB phosphorylation. Overall, sildenafil-based scaffolds were favored for further AD-focused optimization due to better in vivo performance. Building on the concept of multi-target strategies for Alzheimer’s disease, the exploration of dual-acting inhibitors has expanded from PDE5–HDAC combinations to include other phosphodiesterase isoenzymes such as PDE9. The work by Rabal et al. (2019) introduced a novel series of dual-acting compounds targeting both phosphodiesterase 9 (PDE9) and HDACs, aiming to tackle Alzheimer’s disease [102] (Figure 5). This strategy is grounded in the complementary roles of these enzymes in AD. PDE9 controls intracellular cGMP levels associated with cognition and synaptic plasticity, while HDACs, especially HDAC6 and class I isoenzymes, influence gene expression, tau metabolism, and neuronal signaling (Table 4). Compound **10** emerged as a lead, inhibiting HDAC6 (IC_50_ ≈ 40 nM) and PDE9 (IC_50_ ≈ 84 nM) with acceptable brain penetration, low cytotoxicity, and minimal off-target effects, also some hERG activity was noted. Functional cellular assays confirmed its ability to enhance acetylation of histone H3 and α-tubulin and to promote CREB phosphorylation. Building on the success of HDAC–PDE9 dual inhibitors such as compound **10**, researchers have also turned their attention to other neuronal pathways implicated in Alzheimer’s disease. Among these, N-methyl-D-aspartate receptors (NMDARs) have emerged as particularly attractive targets, given their central role in synaptic plasticity and excitotoxicity.

**Figure 6:**
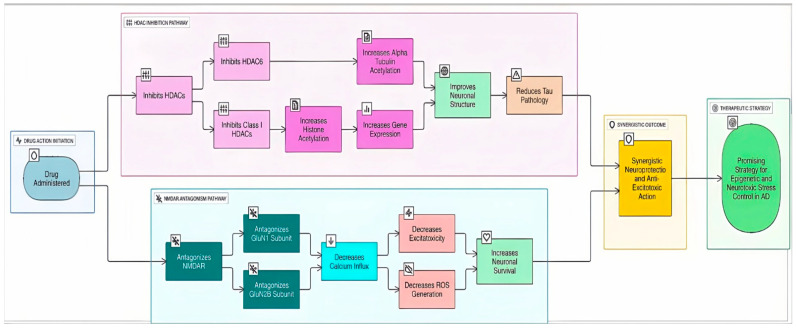
Mechanistic pathways of dual HDAC and NMDAR inhibition for neuroprotective therapy in Alzheimer’s disease.

N-methyl-D-aspartate receptors (NMDARs) are a type of glutamate receptor essential for learning, memory, and synaptic plasticity. In Alzheimer’s disease, changes in NMDAR function and structure are closely linked to cognitive decline, synaptic dysfunction, and neurodegeneration through mechanisms involving excitotoxicity, synaptic dysfunction, and interactions with pathogenic proteins [103]. This makes it another interesting target for a dual-target inhibitor (Figure 6). By combining the memantine scaffold (a known NMDAR antagonist) with zinc-binding groups typical of HDAC inhibitors (such as hydroxamic acid), He et al. (2020) developed multi-target-directed ligands for AD, with compound **11** showing dual HDAC6 inhibition (IC_50_ = 0.18 μM) and NMDAR antagonism (K_i_ = 0.59 μM) [104] (Table 4). For HDAC6, compound **11** formed a monodentate coordination with the catalytic zinc ion and established key interactions with residues such as Gly619 and His610, as well as π–π stacking with Phe620 and His651, similar to known HDAC6 ligands. Docking with NMDAR (GluN1/GluN2B subunits) showed that compound **11** occupied the same binding pocket as memantine, forming hydrogen bonds with critical asparagine residues (Asn616, Asn615), suggesting that the compound retains memantine-like binding despite structural modification. In PC-12 cells, compound **11** increased acetylated α-tubulin, provided neuroprotection against H_2_O_2_ (EC_50_ = 0.94 μM), showed low cytotoxicity (LC_50_ = 16.22 μM in L02 cells), and had favorable BBB permeability (logBB ≈ 0.45), highlighting its potential as a dual-acting neuroprotective AD therapeutic.

**Figure 7:**
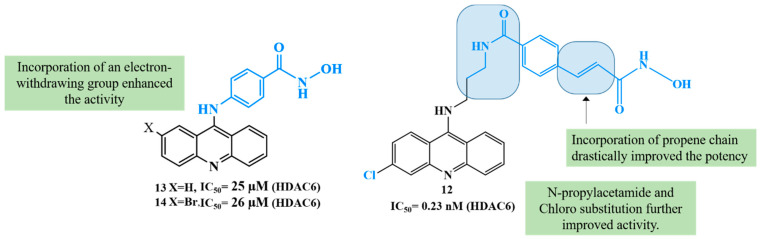
Structure–activity relationship (SAR) of acridine based analogs on HDAC inhibition potency.

Expanding the scope of multi-target strategies in Alzheimer’s disease beyond phosphodiesterase and HDAC modulation. Xu et al. (2020) synthesized a diverse library of tacrine-hydroxamate hybrid molecules aimed at addressing multiple pathological mechanisms involved in Alzheimer’s disease, including HDAC activity, acetylcholinesterase (AChE) inhibition, Aβ aggregation, oxidative damage, and metal ion dysregulation [105]. Among these, compound **12** (Figure 7) (Table 4) was identified as a most promising multi-target potential compound. It showed exceptionally high AChE inhibitory activity with an IC_50_ of 0.12 nM and exhibited strong selectivity over butyrylcholinesterase (BChE), with an IC_50_ of 361.52 nM. Additionally, compound **12** inhibited HDACs from HeLa nuclear extract with a potent IC_50_ of 0.23 nM, suggesting significant epigenetic modulation capacity (Table 2). It also demonstrated antioxidant activity comparable to the standard compound Trolox and showed moderate inhibition (34.2%) and disaggregation (41.24%) of Aβ1–42 oligomers and effective Cu^2+^ chelation (74%), which is relevant in reducing metal-induced oxidative stress and Aβ aggregation. Predictive models also indicated the compound’s potential to cross the blood–brain barrier (BBB), enhancing its drug-likeness for CNS applications in Alzheimer’s disease.

The structure–activity relationship (SAR) presented in the figure (Figure 7) illustrates that introduction of an electron-withdrawing substituent, such as a bromine or chlorine group in compound **14**, on the aromatic ring led to a significant enhancement in activity, likely due to increased electrophilicity and improved metal-binding efficiency of the hydroxamate moiety, while no substitution retains the activity. Furthermore, incorporation of a propene chain linker between the aromatic core and the hydroxamic acid group markedly improved potency in compound **12**, suggesting that the extended conjugation and spatial flexibility facilitate optimal alignment within the enzyme’s active site. Subsequent modification involving the introduction of an N-propylacetamide group, in combination with chloro substitution, resulted in further enhancement of biological activity, indicating that synergistic effects between electronic tuning and optimized steric orientation contribute to improved binding affinity and overall pharmacological efficacy.

**Figure 8:**
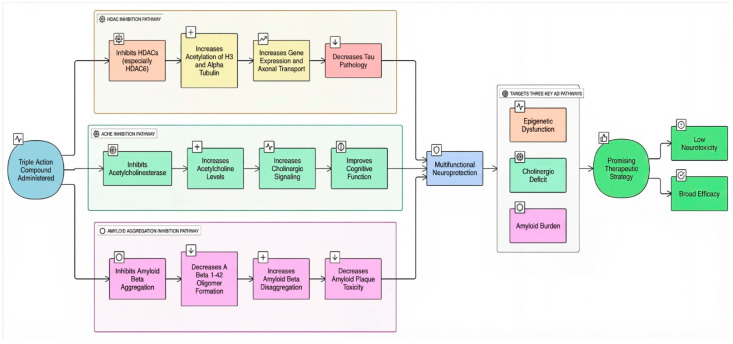
A multifunctional therapeutic approach targeting epigenetic, cholinergic, and amyloid pathways in Alzheimer’s disease.

Triple-action compounds are multifunctional therapeutic agents designed to simultaneously target histone deacetylases (HDACs), acetylcholinesterase (AChE), and amyloid-β aggregation. In Alzheimer’s disease, overactivity of HDACs leads to epigenetic dysregulation and impaired neuronal function, increased AChE activity contributes to cholinergic deficits and cognitive decline, and amyloid-β aggregation drives plaque formation and neurotoxicity. This makes HDACs, AChE, and amyloid-β interconnected targets for a single therapeutic strategy [106,107]. By combining structural motifs capable of HDAC inhibition (such as hydroxamic acids), AChE inhibition (such as carbamate or tacrine-like scaffolds), and anti-amyloid activity, these triple-action compounds aim to restore acetylation balance, enhance cholinergic signaling, and reduce amyloid burden, offering a promising approach for multi-targeted intervention in Alzheimer’s disease (Figure 8). On this account, Tseng and coworkers in (2020), developed acridine-based hydroxamate derivatives as triple-action inhibitors of HDACs, AChE, and Aβ aggregation. Lead compounds **13** and **14** (Figure 7) exhibited potent HDAC6 inhibition (IC_50_ = 0.025 and 0.026 μM, respectively), moderate AChE inhibition (IC_50_ = 0.6–0.8 μM), and strong inhibition and disaggregation of Aβ1–42 oligomers (IC_50_ = 1.1–3.0 μM) [108] (Table 4). Molecular docking studies showed that both compounds engaged in zinc coordination, hydrogen bonding, and hydrophobic interactions within class II HDACs. Biologically, they significantly increased acetylation of histone H3 and α-tubulin, promoted neurite outgrowth in primary hippocampal neurons, and demonstrated low neurotoxicity. Although their PAMPA-BBB permeability was moderate to low, their multifunctional activity and strong safety profile suggest that these acridine derivatives hold promise as therapeutic candidates for AD. This study underscores the potential of acridine scaffolds in developing multifunctional ligands that address epigenetic regulation, cholinergic signaling, and amyloid burden simultaneously, key pathological features in Alzheimer’s disease.

**Table 4:** Multi-target HDAC inhibitors for Alzheimer’s disease therapy.

Key Compounds	Structures	Dual-Target Strategy	HDAC IC_50_	Secondary Target (IC_50_)	Key Features	References
Compound **8**, **9**	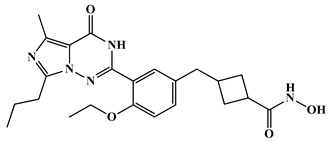 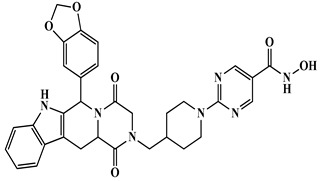	HDAC + PDE5	HDAC6: 7 = 110 nM; 8 = 77 nM	PDE5: 7 = 0.27 nM 8 = 308 nM	Strong dual inhibition; limited solubility and BBB penetration	[101]
Compound **10**	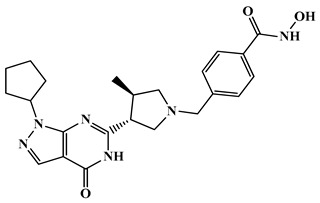	HDAC + PDE9	HDAC6: 40 nM	PDE9: 84 nM	Good brain permeability and target engagement	[102]
Compound **11**	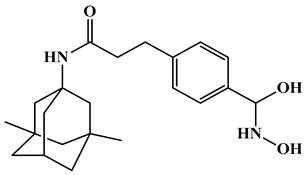	HDAC + NMDAR	HDAC6: 180 nM	NMDAR: K_i_ = 590 nM	Neuroprotective; BBB-permeable; low cytotoxicity	[104]
Compound **12**	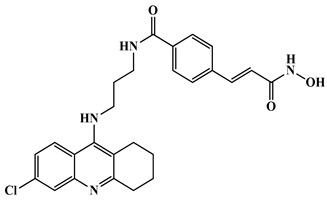	HDAC + AChE + Aβ	HDAC: 0.23 nM	AChE: 0.12 nM	Strong multi-target effects; antioxidant and metal chelation	[105]
Compounds **13**, **14**	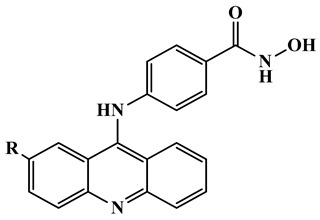 13, R = H, 14, R = Br	HDAC + AChE + Aβ	HDAC6: 12 = 25 µM; 13 = 26 µM	AChE: 12 = 600 nM 13 = 800 nM	Aβ aggregation IC_50_: 1.1–3.0 µM; low neurotoxicity	[108]
Compound **15**	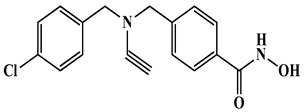	HDAC + MAO-B	HDAC1: 21.4 nM	MAO-B: 99 nM (MAO-A: 9923 nM)	Excellent selectivity; brain-penetrant; in vivo efficacy	[109]
Compounds **16**, **17**	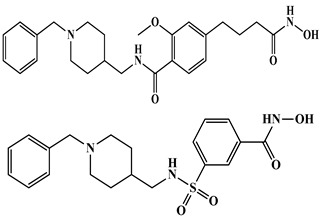	HDAC + AChE	HDAC6 16 = 170 nM; 17 = 450 nM	AChE 16 = 6.89 µM; 17 = 3.22 µM	Antioxidant, metal chelation, Aβ inhibition; stable Zn^2+^ coordination confirmed	[110]
Compound **18**	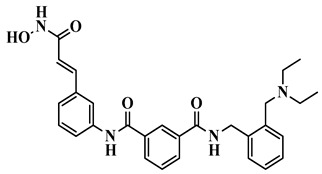	HDAC6 + BChE	HDAC6: 56.7 nM	BChE: 0.3 nM	Highly potent dual inhibitor; neuroprotective and cognitive benefits	[111]

**Figure 9:**
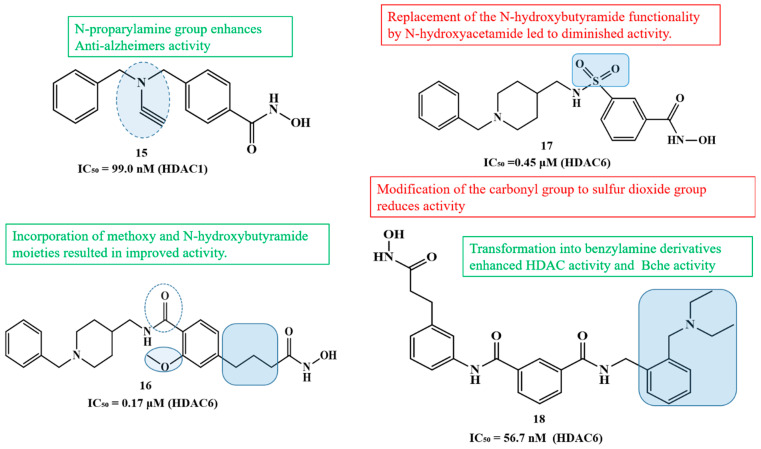
Structure–activity relationship (SAR) showing the effect of various substituents on HDAC inhibition and anti-Alzheimer’s potential of MTDLs.

Building on the concept of multi-target-directed ligands, Yao et al. (2022), designed a series of N-propargylamine-hydroxamic acid/o-aminobenzamide hybrids to simultaneously inhibit HDAC1 and MAO-B, a mitochondrial enzyme responsible for the oxidative deamination of monoamine neurotransmitters, which produces hydrogen peroxide and contributes to neuronal oxidative stress, mitochondrial dysfunction, and neuroinflammation, aiming to address both epigenetic dysregulation and oxidative damage in Alzheimer’s disease [109]. Compound **15** (Figure 9) (Table 4) was the most potent, with IC_50_ = 21.4 nM for HDAC1 and 99.0 nM for MAO-B, showing high selectivity over MAO-A (IC_50_ = 9923 nM; SI = 100.2). It reversed Aβ_1–42_-induced toxicity and reduced ROS in PC12 cells, exhibited excellent BBB permeability, and, in a scopolamine-induced AD mouse model, improved cognitive function in the Morris water maze, outperforming the reference MAO-B inhibitor pargyline.

Expanding on the concept of multifunctional therapeutics, the 2023 study by Qin et al. reports the design, synthesis, and evaluation of novel N-benzyl piperidine derivatives as dual HDAC and AChE inhibitors as multi-target-directed ligands (MTDLs) for potential treatment of AD [110]. These compounds were developed to simultaneously inhibit HDACs and acetylcholinesterase (AChE). Among the synthesized molecules, compounds **16** and **17** (Figure 9) demonstrated potent dual activity, with HDAC IC_50_ values of 0.17 μM and 0.45 μM from HeLa extract and AChE IC_50_ values of 6.89 μM and 3.22 μM, respectively (Table 4). Both compounds also exhibited antioxidant activity, metal-chelating ability, and effective inhibition of Aβ_1–42_ aggregation, which are important for reducing oxidative stress and amyloid burden in AD. Furthermore, in H_2_O_2_-induced neurotoxicity assays, 16 and 17 showed significant neuroprotective effects in PC-12 cells, outperforming donepezil in some cases. Molecular docking revealed that these compounds bind effectively to the active sites of both HDAC6 and AChE, forming stable interactions with key residues and coordinating with Zn^2+^ in HDAC6. The study concludes that these N-benzyl piperidine hybrids, particularly 16 and 17, are promising multi-functional lead compounds with potential for further development as AD therapeutics.

The structure–activity relationship (SAR) illustrated in the figure (Figure 9) demonstrates the impact of various structural and functional modifications, in which the incorporation of methoxy and N-hydroxybutyramide moieties resulted in a notable improvement in activity, highlighting the importance of these functionalities for optimal enzyme interaction in compound **16**. However, replacement of the N-hydroxybutyramide group with N-hydroxyacetamide led to diminished potency in compound **17**, suggesting that chain length and steric factors are critical for maintaining favorable binding. Similarly, modification of the carbonyl group to a sulfur dioxide moiety caused a reduction in activity, indicating the essential role of the carbonyl group in maintaining proper coordination within the active site. On the other hand, transformation of the parent scaffold into benzylamine derivatives significantly enhanced both HDAC and butyrylcholinesterase (BChE) inhibitory activities compound **18**. Furthermore, introduction of an N-propargylamine group conferred enhanced anti-Alzheimer’s activity in compound **15**, possibly through improved interaction with cholinesterase or neuroprotective mechanisms. Collectively, these findings emphasize that fine-tuning of both the zinc-binding domain and terminal amine substituents is crucial for achieving balanced potency and selectivity across multiple biological targets.

Further advancing multi-target approaches in Alzheimer’s disease drug discovery, Lv et al. (2025) designed dual BChE/HDAC6 inhibitors to address the multifactorial pathology of Alzheimer’s disease [111]. Among 18 hybrids, compound **18** (Figure 9) (Table 4) showed potent inhibition of BChE (IC_50_ = 0.3 nM) and HDAC6 (IC_50_ = 56.7 nM), favorable solubility (Log P ≈ 0.15), and low cytotoxicity. Docking confirmed strong dual-target interactions (BChE: Trp82, Tyr332; HDAC6: Zn^2+^, His573, Gly582). In vitro, it increased acetylated α-tubulin and protected neurons from oxidative and glutamate-induced injury. In vivo, compound **18** was safe in acute toxicity tests and improved cognitive performance in scopolamine-induced AD mice (Morris Water Maze, Y-maze, novel object recognition), with effects comparable to donepezil and superior to single-target inhibition, highlighting its potential as a dual-acting neuroprotective AD therapeutic.

Together, these studies highlight the potential of multi-target-directed ligands (MTDLs) as a promising strategy for treating Alzheimer’s disease, which arises from multiple interconnected pathological processes. By combining HDAC inhibition with complementary actions such as PDE modulation, NMDAR receptor antagonism, cholinesterase or MAO-B inhibition, and antioxidant or metal-chelating effects, researchers have developed compounds that can simultaneously influence epigenetic regulation, synaptic function, excitotoxicity, oxidative stress, and cholinergic deficits. Rather than addressing individual disease mechanisms in isolation, these multifunctional agents act across several pathways, offering the possibility of both symptomatic relief and disease modification. The compounds summarized in Table 4 reflects diverse chemical approaches to HDAC modulation, including dual inhibitors, HDAC6-selective agents, and multifunctional hybrids. Many of these designs show added benefits against key Alzheimer’s hallmarks such as amyloid aggregation, tau pathology, neuroinflammation, and oxidative stress. Importantly, optimizing brain penetration, target selectivity, and safety remains essential for clinical translation. Overall, rational polypharmacology through MTDLs represents an innovative and compelling direction for future Alzheimer’s disease therapy.

### 3.2. Parkinson’s Disease

**Figure 10:**
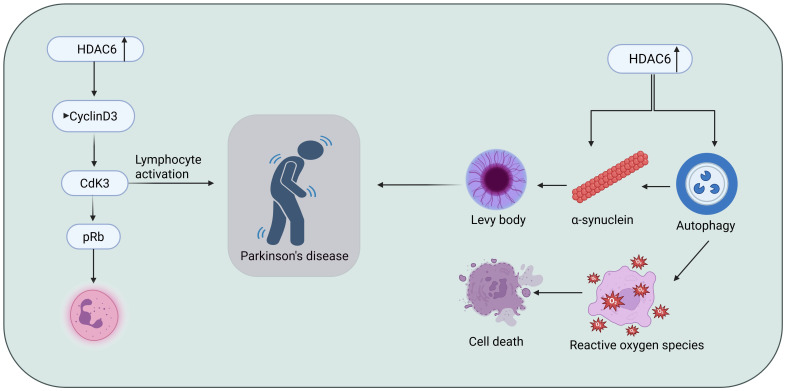
HDAC6-mediated pathways in Parkinson’s disease: intersections between autophagy, α-synuclein aggregation, and immune activation [58]. Solid arrows indicate proposed regulatory or pathological relationships, and upward arrows (↑) denote increased expression or activity. HDAC6 is shown to modulate immune-related pathways and autophagy. α-synuclein processing, contributing to Lewy body formation, oxidative stress, and neuronal cell death in Parkinson’s disease, Pathways are based mainly on experimental and preclinical evidence.

Parkinson’s disease (PD) is a progressive neurodegenerative disorder characterized primarily by motor symptoms such as tremors, bradykinesia, rigidity, and postural instability, alongside non-motor symptoms including cognitive decline and mood disturbances [112]. These clinical features are largely attributed to the degeneration of dopaminergic neurons in the substantia nigra, pars compacta and the accumulation of α-synuclein proteins forming Lewy bodies, leading to insufficient dopamine production [113]. A growing body of evidence suggests that neuroinflammation plays a central role in PD pathogenesis, contributing to neuronal loss through activation of microglia and the release of pro-inflammatory cytokines [114,115]. Among various molecular targets being investigated to modulate neuroinflammation, histone deacetylase 6 (HDAC6) is a cytoplasmic enzyme that deacetylates non-histone proteins, most notably α-tubulin and heat shock protein 90 (HSP90), both of which are critical for cellular transport and protein homeostasis. In the context of Parkinson’s disease (PD), HDAC6 has been increasingly recognized for its involvement in neurodegeneration and inflammation [116]. Its inhibition has been shown to reduce microglial activation and pro-inflammatory cytokine release, key features of PD-associated neuroinflammation [117]. Additionally, HDAC6 plays a role in clearing misfolded proteins like α-synuclein through the autophagy-lysosome pathway, which is often disrupted in PD. By enhancing autophagic flux and stabilizing microtubule dynamics via α-tubulin acetylation, HDAC6 inhibition helps preserve dopaminergic neurons and improve motor function in preclinical PD models [118]. Thus, selective HDAC6 inhibitors, especially those with strong brain-penetrant properties, are being explored as promising therapeutic agents to slow or modify disease progression in Parkinson’s disease (Figure 10).

#### 3.2.1. Multi-Target and Selective HDAC Inhibitors in Parkinson’s Disease Therapy

Recent advances in the development of selective HDAC inhibitors have highlighted compound **19**, a novel brain-permeable compound, as a promising therapeutic candidate for mitigating neuroinflammation in Parkinson’s disease. Liu et al. (2023) [119] developed a brain-permeable HDAC6 inhibitor, compound **19** (Table 5), for neuroinflammation-related diseases, including Parkinson’s. Prior studies showed that pharmacological inhibition of HDAC6 can protect dopaminergic neurons by suppressing inflammatory responses, particularly through downregulating the NLRP3 inflammasome pathway in Parkinson’s disease models [119]. It showed high potency (IC_50_ = 1.8 nM) against HDAC6, with >116-fold selectivity over other HDACs, strong brain penetration (brain/plasma ratio 2.1 at 30 min), and anti-inflammatory effects in LPS-stimulated BV2 cells and mouse models. Docking revealed monodentate zinc coordination, hydrogen bonding (Ser531, His547), π–π stacking (Phe583, Phe643), and favorable van der Waals interactions in the HDAC6 L1-loop. These results support compound **19** as a promising neuroprotective with strong anti-inflammatory activity and HDAC6-targeting candidate for Parkinson’s disease.

In this context, Li et al. (2021) [120] developed compound **20**, a β-carboline-based hydroxamate derivative, designed to target both HDAC6 and mitochondrial pathways, thereby offering complementary mechanisms of neuroprotection beyond inflammation control. Li et al. synthesized a novel HDAC inhibitor, HGC (N-(4-(hydroxycarbamoyl)benzyl)-1-(3,4,5-trimethoxyphenyl)-9H-pyrido[3,4-b]indole-3-carboxamide), and evaluated its neuroprotective effects in cellular and animal models of Parkinson’s disease (PD) [120]. compound **20** exhibited potent inhibition of HDAC6 (IC_50_ ≈ 28 nM) and HDAC1 (IC_50_ ≈ 53 nM), showing greater selectivity and potency compared to SAHA (Table 5). In MPP^+^-treated SH-SY5Y cells and primary dopaminergic neurons, compound **20** significantly improved cell viability, reduced LDH release, preserved mitochondrial membrane potential, and lowered oxidative stress. In MPTP-induced PD mouse models, 20 administration alleviated motor deficits, restored tyrosine hydroxylase (TH) expression in both the substantia nigra and striatum, and protected mitochondrial ultrastructure. Mechanistically, 20 induced acetylation of NDUFV1 at lysine 28, a subunit of mitochondrial complex I, which is normally deacetylated by HDAC6. Overexpression of NDUFV1 mimicked compound **20**’s protective effects, while knockdown of NDUFV1 abolished them, confirming that the HDAC6/NDUFV1 axis mediates compound **20**’s therapeutic action. These findings position compound **20** as a promising HDAC6-targeted agent with disease-modifying potential in PD through mitochondrial protection and epigenetic modulation.

#### 3.2.2. Future Potential for Repurposing HDAC Inhibitors in Parkinson’s Disease

Hiranaka et al. (2018) developed a novel class I-selective histone deacetylase (HDAC) inhibitor, compound **21**, designed to overcome the poor blood–brain barrier (BBB) permeability typical of HDAC inhibitors by incorporating a pyrilamine moiety that targets the pyrilamine-sensitive organic cation antiporter (PYSOCA) [121]. Compound **21** showed selective inhibition of class I HDACs, particularly HDAC1 (IC_50_ = 1.5 µM), and demonstrated significant BBB penetration, with a brain permeability rate (PSBBB) of 42.4 µL/min/g, 3.3 times greater than the reference inhibitor CI-994. Cellular studies confirmed its HDAC-inhibitory activity through increased histone H3K9 acetylation and sustained epigenetic modulation. While not tested directly in Parkinson’s disease (PD) models, this transporter-mediated delivery strategy offers a promising approach for developing CNS-penetrant HDAC inhibitors. Given the role of class I HDACs in neuroinflammation, dopaminergic neuron survival, and epigenetic dysregulation in PD, compounds like this represent a valuable direction for future therapeutic development targeting epigenetic mechanisms in Parkinson’s disease (Table 5). HDAC inhibitors show promising potential in Parkinson’s disease treatment by offering neuroprotective and anti-inflammatory effects. Compounds like compound **21** selectively inhibit HDAC6, helping to reduce inflammation, protect mitochondrial function, and support the survival of dopaminergic neurons. Other inhibitors, such as an HDAC1-selective compound with enhanced brain penetration, target epigenetic dysregulation linked to Parkinson’s. The following inhibitors of Parkinson’s disease are summarized in Table 5. Overall, HDAC inhibitors represent a promising strategy to slow neurodegeneration and alleviate symptoms in Parkinson’s disease.

### 3.3. Huntington’s Disease

Huntington’s disease (HD) is an autosomal dominant genetic neurodegenerative disease and is characterized by progressive degeneration of nerve cells. Huntington’s disease is primarily caused by abnormal expansion of the CAG (cytosine-adenine-guanine trinucleotide) repeat within exon 1 of the *HTT* gene [122]. Huntington’s disease (HtD) was first described in 1872 by Ohio physician George Huntington. In normal individuals, the *HTT* gene has 6 to 35 CAG trinucleotide repeats. However, when the CAG repeats exceed 36, it results in the accumulation of mutant Huntington (mHtt) proteins [123]. These misfolded proteins are involved in diverse cellular disruptions such as dopamine-induced toxicity, oxidative stress, metabolic dysfunction, and defective synaptic and transcriptional functions [124,125]. Huntington’s disease patients demonstrate motor dysfunction such as impairment of walking, impaired coordination, and involuntary jerky movements such as chorea [126]. Recently, the paradigm in the management of Huntington’s disease (HD) has transitioned from the largely symptomatic approach to the treatment modalities that address the disease mechanisms and may impede the disease progression process [127]. In addition to this, histone deacetylase (HDAC) inhibitors are now being considered as promising candidates for disease-modifying approaches. Modifying chromatin structure and regulation of gene expression, HDAC inhibitors may rectify transcriptional imbalance disturbed during Huntington’s disease pathology [128]. This review provides an overview of recent advances in symptomatic treatments and emphasizes the growing interest in mHTT and epigenetic regulator-targeted therapies, such as those involving HDACs.

**Figure 11:**
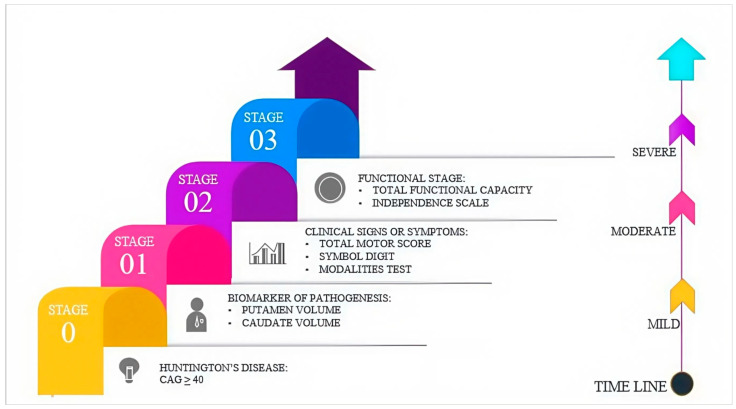
Progressive stages of Huntington’s disease along the clinical timeline.

The Huntington’s Disease Integrated Staging System (HD-ISS) is described in four stages:

• Stage 0: It develops in people with over 39 CAG repeats and may last for decades without any evident structural or clinical abnormalities.
• Stage 1: This marks the beginning of measurable neurodegeneration, detectable through MRI scans. Although still awaiting publication, a recent examination of large datasets suggests that Stage 1 is relatively brief, averaging only 3 to 5 years in duration.
• Stage 2: This stage begins once symptoms become quantifiable on standardized clinical rating scales. It may also include cognitive or motor manifestations of Huntington’s disease and typically lasts for about 7 years.
• Stage 3: This stage is characterized by increased disability, such as difficulties in performing daily activities, again measurable on clinical scales, and usually lasts for approximately 20 years until death [129,130] (Figure 11).

Overall, this indicates that the HD progresses over decades, with the most severe neurodegeneration occurring in Stages 1–2. Understanding this timeline is critical for clinical trial planning and identifying optimal therapeutic windows, though further studies are needed to link these phases to subcellular mechanisms for precision HD treatments.

**Figure 12:**
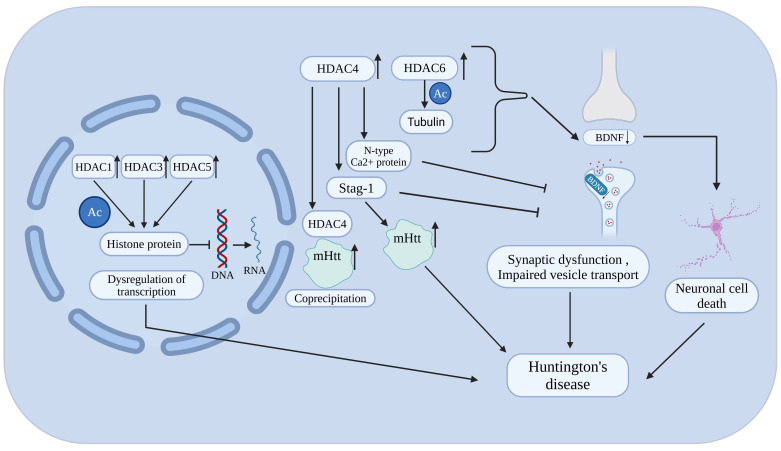
Role of HDAC isoenzyme in Huntington’s disease pathogenesis: crosstalk between transcriptional dysregulation and synaptic impairment [58].

Huntington’s disease (HD) is driven by mutant huntingtin (mHtt), which disrupts transcription and intracellular transport. Increasing evidence indicates that histone deacetylases (HDACs) play an important role in mediating these pathological effects. Altered HDAC activity leads to histone hypoacetylation and repression of genes vital for neuronal survival, including BDNF [131]. One of the most affected pathways involves the downregulation of brain-derived neurotrophic factor (BDNF), a critical neurotrophin required for neuronal maintenance and plasticity. In addition to transcriptional dysregulation, specific HDAC isoenzymes contribute to cellular transport deficits in HD. HDAC6 dysregulation impairs α-tubulin acetylation and axonal transport, further exacerbating neuronal dysfunction [132] (Figure 12).

Collectively, HDAC-mediated epigenetic alterations, impaired axonal transport, and loss of neurotrophic support converge to promote neuronal dysfunction and progressive neurodegeneration in HD. These insights provide a rationale for exploring selective HDAC inhibitors as potential therapeutic strategies to restore transcriptional balance, improve intracellular transport, and enhance neuronal resilience.

#### 3.3.1. HDAC4-Selective Class IIa Inhibitors for Huntington’s Disease Therapy

**Figure 13:**
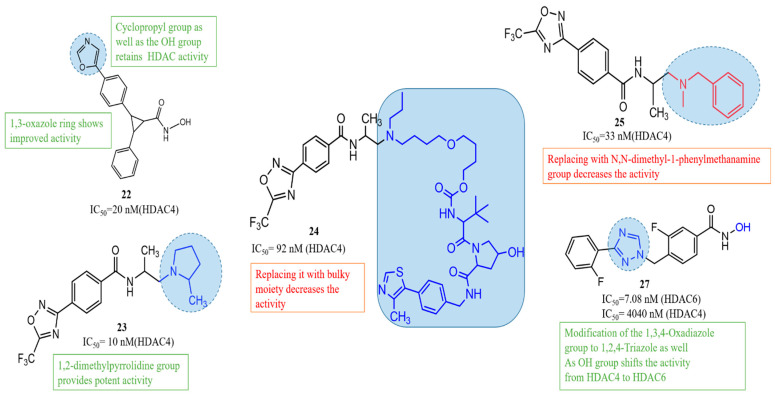
Structure–activity relationship of class IIa histone deacetylase inhibitors highlighting the influence of heterocyclic modifications in Huntington’s disease.

Among the different HDAC classes, class IIa enzymes such as HDAC4 have attracted growing attention due to their involvement in transcriptional repression, neuronal apoptosis, and the progression of neurodegenerative diseases, making them valuable targets for selective inhibitor development [57]. Bürli et al. (2013) designed a novel class of trisubstituted diarylcyclopropane-based hydroxamic acids as selective inhibitors of class IIa histone deacetylases [133]. Using rational design and molecular docking, they optimized three compounds, amongst which compound **22** (Figure 13) in particular was found to be most active among others, to target a unique binding site in HDAC4 not found in other isoenzymes, enhancing specificity. Compound **22** emerged as the most promising, with potent HDAC4 inhibition (IC_50_ = 20 nM) and over 2000-fold selectivity compared to class I and IIb HDACs like HDAC2. Pharmacokinetic studies showed high oral bioavailability (84%) and a brain-to-plasma ratio of 0.2 to 1.0, indicating good central nervous system and muscle exposure. It also displayed strong muscle distribution, making it useful for peripheral HDAC4 studies. Despite some concerns from ADME profiling, such as P-glycoprotein efflux and CYP450 interactions, the researchers addressed these issues through structural modifications, improving stability and reducing metabolic liabilities. Ultimately, compound **22** appears highly selective, offering strong potential for advancing targeted therapies in HD and other neurodegenerative diseases where class IIa HDACs play a pathological role.

Building on the rationale for targeting HDAC-mediated pathways in HD, Stott et al. (2021) [134] developed a series of 5-(trifluoromethyl)-1,2,4-oxadiazole (TFMO)-based inhibitors targeting class IIa histone deacetylases as potential therapeutics for Huntington’s disease, focusing on HDAC4. Compound **23** (CHDI-00484077) (Figure 13) showed potent inhibition of HDAC4 (IC_50_ ≈ 0.01 μM), with similarly low nanomolar potency against HDAC5 (~0.02 μM), HDAC7 (~0.02 μM), and HDAC9 (~0.03 μM), while maintaining >100-fold selectivity over class I and class IIb HDACs [134]. Pharmacokinetic profiling revealed excellent properties, including oral bioavailability, solubility, and strong brain penetration (brain-to-plasma ratio of ~1.16). In vivo studies in mice confirmed target engagement through increased histone H3 acetylation in the brain. The therapeutic efficacy of compound **23** in Huntington’s disease models positions it as a highly selective, brain-penetrant probe for class IIa HDACs, providing a critical platform for dissecting HDAC4’s mechanistic role in neurodegeneration. Building on this foundational work, Macabuag et al. (2022) subsequently developed the first selective HDAC4 protein degraders [42]. The study designed bifunctional PROTACs using a VHL E3 ligase-recruiting strategy to selectively degrade HDAC4 in primary neurons. Among the compounds, compound **24** (Figure 13) (TFMO series) demonstrated potent HDAC4 degradation in HD-relevant models, with a DC_50_ of 1.1 nM in Q175 knock-in mouse cortical neurons. Although the enzymatic HDAC4 IC_50_ for compound **24** was 0.092 µM, it outperformed its parent inhibitor in selective degradation due to favorable protein–protein interactions. These degraders showed minimal impact on HDAC5, 7, and 9, confirming high isoenzyme selectivity. This work highlights HDAC4 as a promising target in HD and provides chemical tools to further explore its role in disease mechanisms and potential therapy.

Overall, the study by Turkman et al. (2022) [135] demonstrates the potential of compound **25** as a highly selective class IIa HDAC inhibitor with both diagnostic and therapeutic applications in neurodegenerative disorders, including Huntington’s disease. Class IIa HDACs, particularly HDAC4 and HDAC5, are dysregulated in HD, contributing to neuronal dysfunction, transcriptional repression, and disease progression [135]. Compound **25** (Figure 13) exhibits nanomolar potency, with IC_50_ values of 33 nM for HDAC4, 50 nM for HDAC5, 88 nM for HDAC7, and 93 nM for HDAC9, while showing minimal activity against class I HDACs, ensuring high selectivity and reduced off-target effects. When radiolabeled with fluorine-18, compound **25** displayed excellent brain penetration, specific binding to HDAC-rich regions such as the cortex and hippocampus, and favorable pharmacokinetics, including rapid blood–brain barrier crossing and high target-to-background ratios. These properties position compound **25** as a dual-purpose therapeutic agent, enabling non-invasive PET imaging of class IIa HDAC expression and potential therapeutic modulation of HD-associated epigenetic dysregulation.

The structure–activity relationship (SAR) analysis of HDAC4-selective inhibitors revealed that substitution with a 1,2-dimethylpyrrolidine moiety markedly enhanced the inhibitory potency, suggesting that the conformational rigidity and steric orientation of this group are favorable for HDAC4 binding in compound **23** (Figure 13). In contrast, replacement of this moiety with bulkier substituents led to a notable decline in activity in compound **24**, highlighting the importance of steric compatibility within the active site. Substitution with a cyclopropyl group was well tolerated and retained HDAC activity in compound **22**, indicating flexibility toward smaller cyclic amines. However, incorporation of N,N-dimethyl-1-phenylmethanamine resulted in a pronounced reduction in inhibitory potency in compound **25**, implying that aromatic bulk at this position disrupts optimal enzyme–ligand interactions. Furthermore, modification of the heterocyclic ring system exhibited a significant impact on selectivity; conversion of the 1,3,4-oxadiazole ring to 1,2,4-triazole altered the selectivity profile in compound **27**, shifting the inhibitory activity from HDAC4 toward HDAC6. Interestingly, transformation of the same ring into a 1,3-oxazole enhanced overall activity in compound **22**, suggesting improved coordination with the zinc-binding domain. Collectively, these findings underscore the delicate interplay between steric effects, electronic features, and ring topology in determining HDAC4 selectivity and potency.

#### 3.3.2. Dual-Target Inhibitors (HDAC + RIPK1)

**Figure 14:**
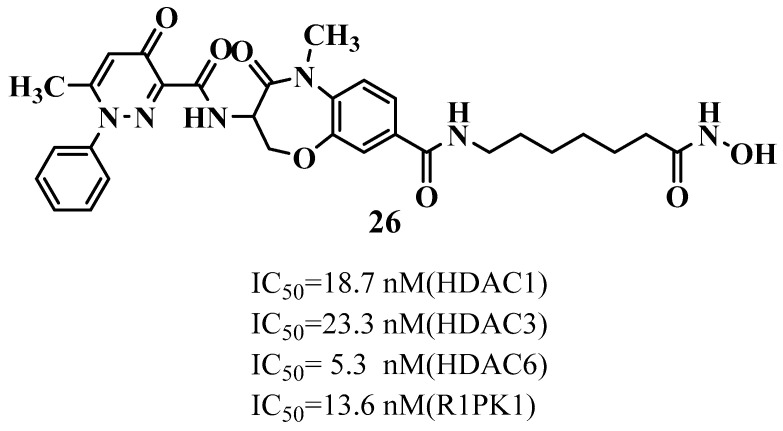
Dual epigenetic and RIPK1-targeting compound in Huntington’s disease treatment.

Receptor-interacting protein kinase 1 (RIPK1) is a serine/threonine kinase that modulates cell fate decisions. It can trigger pro-survival signaling (via NF-κB), apoptosis, or necroptosis depending on cellular context. In neurodegenerative disorders, aberrant activation of RIPK1 contributes to chronic neuroinflammation and neuronal death [136]. The study by Tang et al. (2024) [137] explores the design and evaluation of novel dual inhibitors targeting both receptor-interacting protein kinase 1 (RIPK1) and histone deacetylases (HDACs). These are two key proteins that are involved in neurodegenerative disorders, including Huntington’s disease (HD) [137]. RIPK1 is a critical mediator of necroptosis, a regulated form of inflammatory cell death observed in HD pathology, while HDACs, particularly HDAC6, are involved in epigenetic regulation and cytoskeletal stability. Dysfunctions in both pathways contribute to neuronal loss and neuroinflammation seen in HD. To address this, the researchers synthesized hybrid molecules that combine the inhibitory functions of both RIPK1 and HDACs. Among these, Compound **26** showed potent dual activity, exhibiting nanomolar inhibition of RIPK1 (IC_50_ = 13.6 nM) and HDAC1/3/6 (IC_50_ = 18.7 nM, 23.3 nM, and 5.3 nM, respectively) (Figure 14). Mechanistic docking and molecular dynamics simulations demonstrated strong binding affinity to both RIPK1’s allosteric site and HDAC6’s catalytic domain. The compound also displayed favorable ADMET properties, including good, predicted brain permeability and low toxicity risk. Given that both necroptosis and HDAC dysregulation are involved in Huntington’s disease, this dual-target approach holds therapeutic potential for mitigating neuronal death and inflammation in HD. Thus, Compound **26** emerges as a promising lead for further development in neurodegenerative disease therapies, including Huntington’s disease.

#### 3.3.3. HDAC6-Selective Class IIb Inhibitors for Huntington’s Disease Therapy

HDAC6 plays a pivotal role in the maintenance of cytoskeletal structure, protein homeostasis, and intracellular transport, all of which are disrupted in Huntington’s disease. Considering its involvement in neurodegeneration, several research groups have developed selective HDAC6 inhibitors. Kong et al. (2023) developed a new class of N-benzyltriazolyl-hydroxamate derivatives targeting HDAC6 [138]. Compound **27** (Figure 13), (3-fluoro-4-((3-(2-fluorophenyl)-1H-1,2,4-triazol-1-yl)methyl)-N-hydroxybenzamide) emerged as the lead candidate, exhibiting potent HDAC6 inhibition (IC_50_ = 7.08 nM) and 42-fold selectivity over HDAC1, thereby reducing the likelihood of off-target effects in the brain. Structure-activity relationship (SAR) studies and molecular docking confirmed that the fluorinated triazole scaffold of compound **27** enhances binding specificity to HDAC6, particularly through favorable π–π interactions and hydrogen bonding with key residues like Ser531. In cell-based assays using SH-SY5Y and HeLa cells, compound **27** selectively increased α-tubulin acetylation, a hallmark of HDAC6 inhibition, without significantly affecting histone H3 acetylation, which is regulated by other HDACs. Notably, compound **27** demonstrated a much higher therapeutic index (TI = 108.5) than the broad-spectrum HDAC inhibitor SAHA, suggesting better safety for long-term use in neurons. Overall, compound **27** represents a strong candidate for further development in HD therapy, particularly for restoring protein homeostasis and cellular function.

**Figure 15:**
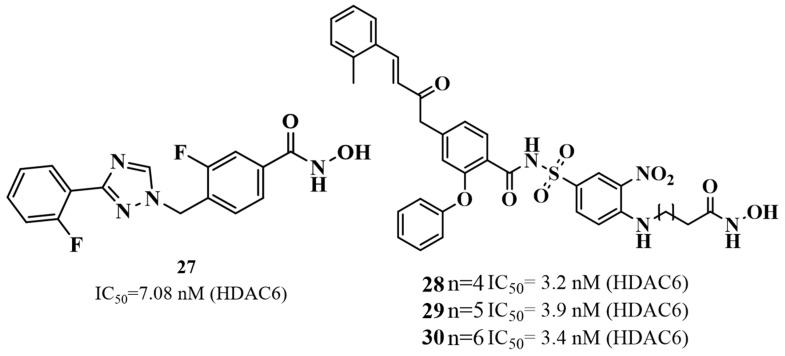
HDAC6 inhibitors in Huntington’s disease treatment.

Recognizing HDAC6’s importance in Huntington’s disease, Zhang et al. (2022) developed and optimized a novel series of selective HDAC6 inhibitors derived from a previously known dual Bcl-2/HDAC6 scaffold [139]. The team focused on refining compounds to better fit HDAC6’s large binding pocket by introducing structural modifications such as extended alkyl linkers and aromatic capping groups. This led to the identification of three lead compounds, **28**, **29**, and **30**, which showed potent HDAC6 inhibition (IC_50_ = 3.2–3.9 nM) and selectivity over HDAC1 (up to 38.7-fold). SAR study indicates that all three compounds show similar HDAC6 inhibitory potency (IC_50_ = 3.2–3.9 nM), suggesting that this range provides optimal spacing between the zinc-binding hydroxamate and the cap region. The linker length of *n* = 4 gives the best activity (IC_50_ = 3.2 nM), implying slightly improved positioning of the hydroxamate in the catalytic pocket, while further extension (*n* = 5 or 6) slightly decreases potency, likely due to increased flexibility and less favorable binding orientation (Figure 15). Thus, *n* = 4 is the optimal linker length for maximum HDAC6 inhibition in this series. Although mainly evaluated in multiple myeloma cell lines, these compounds exhibited low cytotoxicity in normal cells and retained strong HDAC6 specificity. Given HDAC6’s role in HD and its ongoing clinical investigation, these compounds hold promise for future application in restoring cellular balance in neurodegenerative conditions.

### 3.4. S-Triazine-Based HDAC Inhibitors in Neurological Disorders

Triazines are heteroaromatic compounds with the molecular formula C_3_H_3_N_3_, consisting of a six-membered ring containing three carbon and three nitrogen atoms. They serve as versatile structural frameworks in both medicinal and industrial chemistry, forming the basis of numerous derivatives with applications as herbicides, anticancer, antimicrobial, anti-ulcer agents, resins, and pharmaceuticals [140]. Triazines exist in three isomeric forms: 1,2,3-triazine, 1,2,4-triazine, and 1,3,5-triazine (s-triazine), with the latter being the most widely utilized in commercially available agents. s-Triazine (1,3,5-triazine) is an aromatic, planar heterocyclic ring stabilized by delocalized π-electrons, similar to benzene but more electron-deficient due to nitrogen substitution. This isoenzyme features three nitrogen atoms symmetrically arranged at alternating positions, making it chemically stable and highly functionalizable. Substitution typically occurs at the 2, 4, and 6 positions, allowing extensive derivatization and scaffold modification [141]. The electron-deficient nature of s-triazine enables hydrogen bonding and metal coordination, contributing to its wide utility in drug–target interactions, catalysis, and coordination chemistry. Notably, several therapeutic agents such as Altretamine and Tretamine, which are used in cancer treatment, are based on the s-triazine framework [142]. Collectively, these properties establish s-triazine as a privileged scaffold in drug design and industrial chemistry, underpinning its continued importance in both basic and applied research.

**Figure 16:**
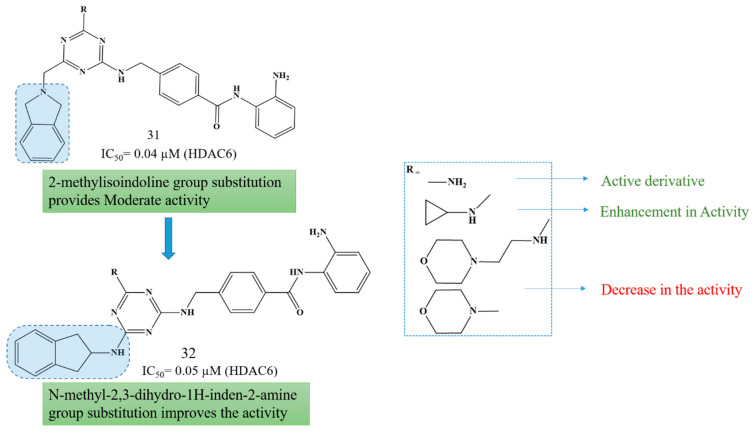
SAR of S-triazine-containing compounds.

Building upon this scaffold, Paquin et al. (2008) reported the design, synthesis, and biological evaluation of 4-[(s-triazin-2-ylamino)methyl]-N-(2-aminophenyl)-benzamides as a novel class of histone deacetylase (HDAC) inhibitors with potent anticancer potential [143]. Among the various heteroaromatic scaffolds synthesized, the s-triazine derivatives exhibited the most promising compound **31** exhibited good HDAC1 inhibitory activity(IC_50_ = 0.04–0.2 µM) from which compound **31** exhibited HDAC1 inhibitory activity (IC_50_ = 0.04 µM) and compound **32** exhibited HDAC1 inhibitory activity (IC_50_ = 0.05 µM). The compounds demonstrated strong antiproliferative effects against HCT116 human colon cancer cells, with minimal cytotoxicity toward normal HMEC cells. Structure–activity relationship (SAR) studies revealed that indanyl-2-amino and isoindolinyl substitutions enhanced HDAC1 inhibition, while amino or alkylamino groups at the R position further improved potency (Figure 16). The s-triazine compounds showed selectivity toward class I HDACs (HDAC1, 2, and 3), induced histone H4 hyperacetylation and p21^WAF1,Cip1^ expression, and displayed significant in vivo antitumor efficacyin HCT116 xenograft models with minimal systemic toxicity. Collectively, these results highlight s-triazine-based benzamides as potent, selective HDAC class I inhibitors with strong therapeutic promise for cancer treatment.

**Figure 17:**
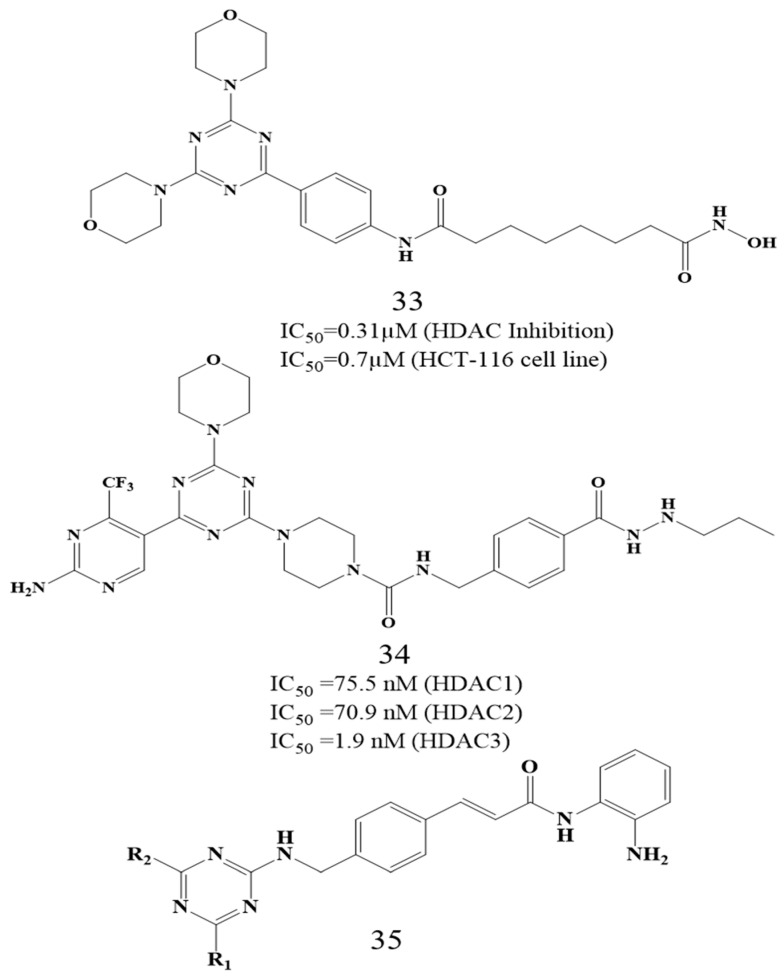
S-Triazine derivatives showing histone deacetylase (HDAC) inhibition.

Building upon this scaffold, Zhao et al. (2014) [144] describe the synthesis and biological evaluation of 1,3,5-triazine-based hydroxamic acids as potent histone deacetylase (HDAC) inhibitors with anticancer activity. Tested in HCT-116 (colon), MCF-7 (breast), and HeLa (cervical) cell lines, most compounds showed strong HDAC inhibition and antiproliferative effects, with compound **33** demonstrating the highest potency (HDAC IC_50_ = 0.31 μM; HCT-116 IC_50_ = 0.7 μM) [144]. Mechanistic studies revealed that compound **33** induced apoptosis and triggered G2/M cell cycle arrest in HCT-116 cells in a concentration-dependent manner, confirming its therapeutic potential. Beyond oncology, these findings are highly relevant to neurological disorders, since epigenetic dysregulation via aberrant histone deacetylase activity is implicated in diseases such as Alzheimer’s, Parkinson’s, and Huntington’s disease. HDAC inhibitors have been shown to improve synaptic plasticity, memory formation, and neuronal survival in preclinical models (Figure 17). Thus, the triazine-based hydroxamic acid scaffold, particularly compound **33**, may serve as a valuable lead for developing next-generation neuroepigenetic therapeutics. Future work could optimize these molecules for blood–brain barrier penetration, enhance isoenzyme selectivity to minimize systemic toxicity, and test efficacy in models of neurodegeneration, potentially paving the way for novel disease-modifying treatments for a broad range of neurological conditions.

Hou et al.’s (2025) [145] study highlights the role of HDAC inhibition in the development of hydrazide-based dual PI3K/HDAC inhibitors for cancer therapy. The lead compound, **34**, showed potent and selective inhibition of HDAC1-3 (IC_50_ values of 75.5 nM, 70.9 nM, and 1.9 nM, respectively) [145]. Functionally, it increased acetylation of histones H3 and H4, confirming effective HDAC blockade. Compound **34** more strongly induced apoptosis by reducing anti-apoptotic Bcl-xL, increasing DNA damage marker γH2AX, and activating caspase-3. In mantle cell lymphoma cells, compound **34** induced ~47% apoptosis at 0.5 μM, demonstrating that HDAC inhibition synergizes with PI3K blockade to promote potent pro-apoptotic activity (Figure 17). Thus, selective HDAC 1-3 inhibition was a key driver of the enhanced anticancer efficacy of this dual-target design. Strategies such as reducing molecular polarity, optimizing lipophilicity, and employing prodrug approaches or nanocarrier delivery systems could enhance CNS penetration. Since aberrant HDAC activity and PI3K/AKT/mTOR dysregulation are implicated in neurodegenerative disorders like Alzheimer’s, Parkinson’s, and Huntington’s disease, a BBB-permeable analog of compound **34** could represent a novel dual-target therapeutic. Thus, adapting compound **34** beyond oncology into CNS-directed therapy could open an exciting frontier in the treatment of neurodegeneration.

Beyond clinically established HDAC inhibitors, recent compound library explorations have identified a diverse set of small molecules with promising epigenetic modulatory potential. A SciFinder-based search retrieved 51 structurally related compounds, most of which belong to the N-(2-aminophenyl)-substituted 1,3,5-triazinyl-benzamide class (e.g., CAS No. 503039-90-5, 503040-90-2, 503041-28-9), i.e., compound code 35 is the core scaffold for all 51 compounds (Figure 17). These derivatives exhibit molecular weights in the range of 347–472 Da and predicted densities between 1.31–1.45 g/cm^3^, aligning with drug-like properties favorable for central nervous system (CNS) activity. Notably, most compounds demonstrate strongly basic pKa values (~13), consistent with their ability to interact with zinc-dependent HDAC catalytic sites. Their structural motifs, including benzamide cap groups and triazinyl zinc-binding domains, closely mirror the canonical HDAC inhibitor pharmacophore, suggesting potential for both pan- and isoenzyme-selective inhibition. Although experimental characterization remains limited, these molecules offer a valuable scaffold for further optimization toward brain-penetrant, HDAC6- and HDAC11-selective inhibitors [146]. The clinical application of a number of HDAC inhibitors in neurodegenerative diseases has been restricted, despite encouraging preclinical results. While obtaining efficient blood–brain barrier penetration continues to be a significant pharmacokinetic challenge, broad-spectrum HDAC inhibitors frequently display dose-limiting toxicities and poor isoenzyme selectivity, which limit their long-term therapeutic utility. These problems have made HDAC inhibitors less successful in clinical settings, highlighting the need for future HDAC-based treatments to have better target specificity and safety profiles.

## 4. HDAC Inhibitors in the Treatment of Neuro-Oncological Disease

Neuro-oncological disorders encompass a broad spectrum of tumors that originate in or affect the central and peripheral nervous systems, including both primary tumors that affect the brain and spinal cord, as well as secondary (metastatic) tumors that spread from other organs. The clinical manifestations of these conditions vary depending on the tumor’s type, location, and growth rate, with common symptoms including seizures, headaches, cognitive deficits, and motor impairments [147]. Also, it depends on patient-related biological factors, including sex and age, which may influence the efficacy and tolerability of HDAC inhibitors in neuro-oncological diseases. Age-dependent variations in HDAC expression and epigenetic regulation have been reported in the human brain, which may affect therapeutic responses in adult versus pediatric patients [66]. In addition, sex-specific differences in glioma biology and clinical outcomes indicate that male and female patients may respond differently to HDAC inhibitor-based therapies [148]. These observations underscore the importance of considering age and sex as biological variables when evaluating HDAC inhibitor treatments and designing future neuro-oncological clinical studies.

These disorders require a multidisciplinary treatment approach involving surgery, radiation, chemotherapy, and increasingly, molecular-targeted therapies such as histone deacetylase inhibitors (HDACis) [149]. Gliomas arising from glial cells are the most prevalent primary brain tumors and include glioblastomas, astrocytomas, and oligodendrogliomas, while meningiomas are usually benign tumors of the meninges. These tumors vary in origin, behavior, and prognosis [150,151]. Medulloblastomas are malignant pediatric cerebellar tumors, and primary CNS lymphomas are rare but aggressive. Schwannomas, including acoustic neuromas, arise from peripheral nerve cells. Other notable types are pituitary adenomas, spinal cord tumors, and brain metastases from cancers like lung or breast carcinoma [152]. These tumors vary widely in their behavior, prognosis, and responsiveness to treatment, with only select types, particularly those with epigenetic dysregulation, showing potential sensitivity to emerging therapies such as histone deacetylase inhibitors (HDACis) [59].

### 4.1. Development and Evaluation of HDAC Inhibitors for Glioblastoma Management

Glioblastoma (GBM) is the most aggressive primary malignant brain tumor, arising from astrocytic glial cells and accounting for 48.6% of malignant CNS tumors [130]. Classified as WHO grade IV, GBM exhibits rapid growth, invasiveness, and poor prognosis, with median survival rarely exceeding one year despite surgery, radiotherapy, and temozolomide [153]. Lower-grade tumors (Grade I) grow slowly and are often curable with surgery, while Grade II tumors infiltrate surrounding tissue, increasing recurrence risk and need for adjuvant therapy [154]. Higher-grade tumors (III–IV) show rapid growth and atypia, requiring aggressive multimodal treatment. Grade IV glioblastoma, in particular, has a poor prognosis, with median survival rarely exceeding one year despite intensive therapy [155,156] (Table 6). The incidence ranges from 0.59–5 per 100,000 and is rising due to aging populations and improved diagnostics [157]. Regardless of advancements in understanding biology, these insights have yet to lead to major improvements in treatment options or patient outcomes. Malignant gliomas make up only 1–2% of all tumors; they are especially dangerous and debilitating [158]. These tumors grow invasively, resist current treatments, and affect the delicate environment of the central nervous system (CNS), making them very difficult to treat [159]. The primary focus is on the 90–95% of glioblastomas lacking IDH mutations (IDH-wt), which are associated with a poorer prognosis [160]. Recent studies have even found similarities between gliomas and specific types of brain stem or glial progenitor cells, suggesting a deeper biological connection between normal neurodevelopment and tumor biology [159]. Various experimental drugs and targeted therapies are currently being investigated, many showing promise in preclinical or early clinical studies [161]. Epigenetic dysregulation, particularly aberrant HDAC activity, is common in GBM, making HDAC inhibitors promising candidates that can modulate signaling pathways, tumor stem-like cells, and immune responses [162].

**Figure 18:**
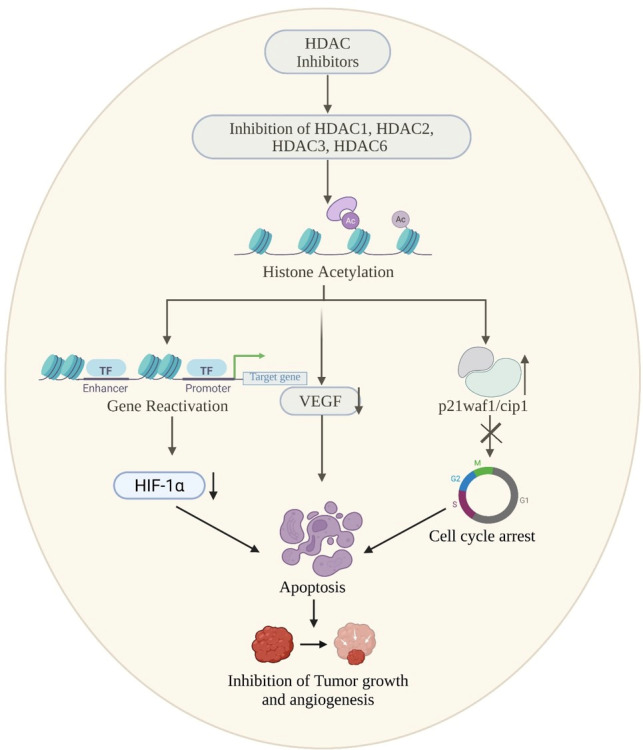
Mechanistic overview of HDAC inhibitor-mediated antitumor effects in glioblastoma [58].

Histone deacetylase (HDAC) inhibitors represent a promising class of epigenetic therapeutics for glioblastoma, acting through multiple mechanisms that disrupt tumor growth and survival [149]. These agents inhibit the enzymatic activity of HDAC isoenzymes, particularly HDAC1, HDAC2, HDAC3, and HDAC6, which are frequently overexpressed in glioblastoma and contribute to transcriptional repression by removing acetyl groups from histone tails [163]. Inhibition of HDACs leads to the accumulation of acetylated histones, resulting in chromatin relaxation and enhanced accessibility of transcription factors to DNA regulatory regions, such as enhancers and promoters [164] (Figure 18). This change facilitates the reactivation of silenced tumor suppressor genes and regulatory elements, including pathways involving HIF-1α and VEGF. Reactivation of HIF-1α contributes to apoptotic signaling, while suppression of VEGF expression limits angiogenesis, reducing the tumor’s ability to sustain its vascular supply [165]. Additionally, HDAC inhibition upregulates the expression of cell cycle regulatory proteins such as p21^WAF1,Cip1^, which halts cell cycle progression at the G1 phase, further limiting glioblastoma proliferation [166]. The combined effects of gene reactivation, apoptosis induction, inhibition of angiogenesis, and cell cycle arrest synergistically contribute to the suppression of tumor growth and progression, positioning HDAC inhibitors as attractive candidates in the therapeutic landscape of glioblastoma.

### 4.2. Multi-Target and Selective HDAC Inhibitors in Glioblastoma Therapy

**Figure 19:**
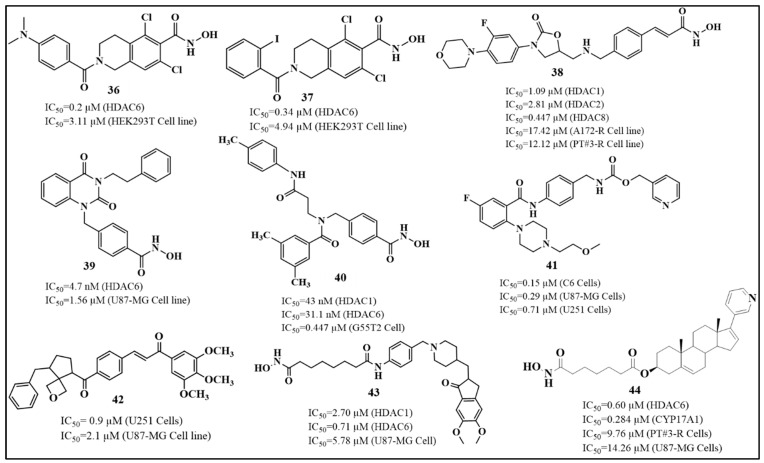
Structural representation of HDAC-selective inhibitors illustrating key pharmacophoric variations for glioblastoma.

In this 2023 study, Khetmalis et al. synthesized 31 novel tetrahydroisoquinoline (THIQ)-based hydroxamate derivatives (B1–B31), aiming to selectively inhibit HDAC6, a class IIb HDAC implicated in cancer progression. Among them, compounds **36** and **37** demonstrated potent HDAC6 inhibition with IC_50_ values of 0.2 μM and 0.34 μM, respectively, and showed negligible activity against HDAC4 and HDAC8. Compound **36** emerged as the lead candidate, exhibiting significant cytotoxicity in U87-MG glioblastoma cells (IC_50_ = 3.11 μM) and MCF-7 breast cancer cells, while showing limited toxicity toward non-cancerous HEK293T cells (IC_50_ = 56.86 μM) [167]. Mechanistic studies revealed induction of apoptosis, caspase-3/7 activation, PARP cleavage, and G1-phase cell cycle arrest. Western blot analysis confirmed selective HDAC6 inhibition via increased α-tubulin acetylation without affecting histone H3 acetylation. Molecular docking showed strong binding to the HDAC6 active site (PDB: 5EDU) through bidentate zinc coordination and π–π interactions (Figure 19). Building on the promising HDAC6-targeted profile of compound **36**, researchers have sought additional strategies to exploit HDAC modulation in glioblastoma therapy. Recognizing the limitations of conventional chemotherapy, particularly temozolomide resistance in GBM, efforts have turned toward developing brain-penetrable HDAC inhibitors with improved pharmacokinetics and isoenzyme selectivity. Such approaches aim not only to inhibit tumor growth but also to enhance epigenetic regulation and overcome chemoresistance mechanisms.

In line with this rationale, Chen et al. (2025) presented an approach to combat glioblastoma multiforme (GBM) by repurposing the antibiotic linezolid into a brain-penetrable histone deacetylase (HDAC) inhibitor, named compound **38** [168]. The rationale stemmed from linezolid’s well-established ability to cross the blood–brain barrier (BBB), with penetration rates reaching over 70% in CNS infections. By structurally modifying linezolid, the researchers introduced an HDAC-inhibitory moiety to target epigenetic mechanisms associated with TMZ resistance, such as the overexpression of HDACs and DNA repair proteins like RAD51 (Figure 19). Among the series of synthesized compounds, compound **38** exhibited broad and potent inhibitory activity across multiple HDAC isoenzymes, with IC_50_ values of 1.09 µM for HDAC1, 0.106 µM for HDAC6, and 0.447 µM for HDAC8. This profile indicates strong inhibition of both class I and class II HDACs, contributing to increased acetylation of histones H3 and H4 and subsequent disruption of DNA repair mechanisms in glioblastoma (GBM) cells. Notably, Compound **38** showed cytotoxicity, with IC_50_ values of 17.41 ± 1.21 μM in A172-R cells and 12.12 ± 1.48 μM in PT#3-R TMZ-resistant GBM cells. Importantly, it was selectively exhibiting much higher IC_50_ values in primary astrocytes (143.1 ± 7.9 μM) and neurons (65.93 ± 4.0 μM), indicating limited toxicity to normal brain cells. Mechanistically, the compound promoted histone acetylation, downregulated DNA repair genes such as *RAD51* and *CtIP*, and induced DNA damage, G2/M arrest, and apoptosis. In an in vivo study, it reduced tumor burden and improved survival in orthotopic GBM mouse models without neurotoxicity, supported by a favorable pharmacokinetic profile (brain/plasma ratio of 1.27 and T_1_/_2_ = 12.04 h).

Liu et al. (2019) [169] reported the design and characterization of compound **39**, a highly selective quinazoline-2,4-dione-based HDAC6 inhibitor for glioblastoma therapy. Compound **39** [169] exhibited potent HDAC6 inhibition (IC_50_ = 4.7 nM) with >2000-fold selectivity over class I HDACs and demonstrated superior activity compared to tubastatin A and SAHA. In glioblastoma U87-MG cells, it suppressed proliferation (IC_50_ = 1.56 μM), inhibited migration, and induced autophagic cancer cell death through blockade of autophagosome lysosome fusion [168]. Mechanistically, compound **39** showed PROTAC-like properties by promoting proteasomal degradation of HDAC6, leading to p62 accumulation, reduced STAT3 phosphorylation, and downregulation of PD-L1, thereby enhancing CD8^+^ T-cell-mediated antitumor immunity (Figure 19). In an in vivo study, compound **39** significantly inhibited tumor growth in U87-MG xenograft models (>80% TGI) without observable toxicity or body weight loss and induced strong immune responses, evidenced by elevated IL-2 and IFN-γ and decreased IL-6 levels. Collectively, these results identified compound **39** as a brain-tumor-targeting, dual-function anti-GBM candidate acting through autophagy modulation and immune activation, supporting its further preclinical development.

Consistent with this approach, Reßing et al. (2019) developed a novel series of β-peptoid-capped histone deacetylase inhibitors (HDACi) specifically designed to target HDAC1 and HDAC6, two enzymes strongly associated with the progression of glioblastoma and neuroblastoma [170]. They synthesized 11 compounds, all featuring a β-peptoid structure that provided improved resistance to proteolytic degradation and enhanced cellular uptake compared to conventional peptide-based inhibitors. These compounds demonstrated strong nanomolar inhibition of HDAC6 (IC_50_: 10–31 nM), outperforming the reference drug vorinostat (IC_50_: 34 nM). Many also effectively inhibited HDAC1, with compound **40** showing the best profile, acting as a potent, non-selective dual inhibitor with HDAC1 IC_50_: 43.0 nM and HDAC6 IC_50_: 31.1 nM. Compound **40** exhibited strong antiproliferative effects in five cancer cell lines, with submicromolar IC_50_ values of 0.38 μM (CHP-134), 0.21 μM (IMR-32), 0.22 μM (SK-N-AS), 0.67 μM (NB-1), and 0.37 μM (G55T2 glioblastoma), clearly outperforming vorinostat, whose IC_50_ ranged from 0.62 to 2.71 μM (Figure 19). Compound **40** triggered a significant rise in apoptotic cell populations in glioblastoma, increasing the sub-G0 phase from 3.5% at 1 μM to 23.3% at 3 μM, closely resembling vorinostat’s activity. Higher concentrations of compound **40** also disrupted the cell cycle by reducing G0/G1 phase cells and increasing apoptotic fractions. Molecular docking revealed that both cis and trans-rotamers of compound **40** successfully engaged the active sites of HDAC1 and HDAC6, supporting the observed biological data. Altogether, this work presents compound **40** as a highly effective dual HDAC1/6 inhibitor with superior anti-cancer activity, laying the groundwork for its development as a next-generation epigenetic therapy for aggressive brain tumors such as glioblastoma and childhood neuroblastoma.

Both Lu et al. (2022) [171] and Zhu et al. (2023) [172] developed novel, HDAC-based compounds showing strong cytotoxicity and blood–brain barrier (BBB) permeability against glioblastoma multiforme (GBM) cell lines. Lu et al. identified compound **41** (Figure 19), a piperazine-based benzamide derivative, with potent cytotoxicity (IC_50_ = 0.15 μM in C6, 0.29 μM in U87-MG, and 1.25 μM in U251 cells) and superior efficacy to temozolomide. Despite exhibiting weak HDAC inhibitory activity, it induced apoptosis and G0/G1 cell cycle arrest by modulating the p16INK4a–CDK4/6–pRb pathway, achieving 70.5% tumor inhibition in vivo without toxicity. Similarly, Zhu et al. reported compound **42** (Figure 19), a 3-oxetanone-derived spirocyclic compound, showing strong antiproliferative effects (IC_50_ = 0.9 μM in U251 and 2.1 μM in U87 cells) by activating the SIRT1/p53 apoptotic pathway while leaving HDAC activity largely unaffected, and demonstrated good BBB permeability and in vivo tumor suppression with no observable toxicity. Together, both studies highlight compound **41** and compound **42** as brain-penetrant, well-tolerated, and potent anti-GBM agents that achieve strong cellular effects despite weak HDAC inhibition, acting primarily through cell cycle and apoptosis regulation, and offer promising leads for further preclinical development.

A dual-targeting approach was further exemplified by Nepali et al. (2023) for treating glioblastoma multiforme, with a focus on overcoming resistance to temozolomide [173]. The researchers designed a new class of donepezil-derived hydroxamic acids that simultaneously activate the sigma-1 receptor (Sig-1R) and inhibit histone deacetylases (HDACs). By incorporating the pharmacophore of donepezil, a known CNS-penetrant and Sig-1R agonist, into an HDAC inhibitory scaffold, they synthesized several derivatives with chemically diverse linkers. Among these, compound **43** showed the most potent anti-GBM activity. Compound **43** achieved significant cytotoxicity in U87MG glioma cells (IC_50_ = 5.78 μM) and was markedly more effective than both donepezil and the reference HDAC inhibitor SAHA. It was also highly effective in killing TMZ-resistant GBM cells and suppressing glioma stem cell (GSC) viability (Figure 19). The compound promoted G2/M cell cycle arrest, followed by subG1 accumulation and apoptosis, and modulated key regulatory proteins, such as increasing acetylation of histone H3, tubulin, and Sig-1R, while reducing levels of PCNA and Sp1. Enzyme inhibition assays revealed that compound **43** selectively inhibited HDAC isoenzymes, with particularly strong activity against HDAC6 (IC_50_ = 2.70 nM) and HDAC2 (IC_50_ = 0.71 μM), along with notable inhibition of HDAC1 and HDAC8. In animal models, compound **43** significantly extended survival in mice with TMZ-resistant U87MG tumors, validating its in vivo potential and therapeutic relevance.

In a 2024 study, Sharma et al. [174] developed a series of abiraterone-installed hydroxamic acids as dual CYP17A1–HDAC6 inhibitors to overcome temozolomide (TMZ) resistance in glioblastoma (GBM). Among the synthesized analogs, compound **44** (Figure 19), featuring an ester linkage and a five-methylene spacer, was identified as the most active, inhibiting CYP17A1 with an IC_50_ of 0.284 μM and HDAC6 with an IC_50_ of 0.60 μM, while strongly increasing α-tubulin acetylation [174]. It showed selective cytotoxicity toward TMZ-resistant GBM cells (IC_50_ = 9.76 μM in Pt#3-R, 12.23 μM in A172-R, and 14.26 μM in U87MG-R) compared to negligible toxicity in normal astrocytes (IC_50_ = 281.6 μM). Mechanistic studies revealed induction of apoptosis, sub-G1 accumulation, suppression of recurrence-associated genes (CDH23, LRMP, LCP1, IFI44L, SLC25A27, NPY1R, SLITRK1), downregulation of Rad51 and antioxidant proteins (SOD1/2, GPX1/4), and enhanced ROS production leading to DNA double-strand breaks (γH2AX). Molecular docking demonstrated stable binding of compound **44** to wild-type CYP17A1 (binding energy −173.9, hydrogen bond contribution −41.5) and HDAC6 (binding energy −145.9, hydrogen bond contribution −34.5), with weaker interactions in mutant proteins, confirming specificity. In vivo, compound **44** significantly inhibited tumor growth in TMZ-resistant xenograft and orthotopic GBM mouse models, extending survival from 28.5 to 45.5 days, with no observable weight loss or systemic toxicity. Altogether, this work establishes compound **44** as a first-in-class dual CYP17A1/HDAC6 inhibitor that integrates epigenetic and metabolic disruption to counteract TMZ resistance and GBM recurrence.

Overall, these findings establish HDAC inhibitors, both selective and multifunctional, as promising agents for overcoming resistance and enhancing efficacy in glioblastoma therapy, especially when combined with BBB permeability, ROS modulation, or additional signaling targets.

## 5. Future Perspective

Despite significant advancements in the understanding of the epigenetic functions of histone deacetylases in neurological disorders, there are still a number of obstacles to overcome before HDAC-based treatments can be successfully implemented in clinical settings. The development of isoenzyme-selective HDAC inhibitors with enhanced blood–brain barrier penetration and decreased off-target toxicity should be the top priority for future research. Simultaneously, new tactics like proteolysis-targeting chimeras (PROTACs) present a viable method for the targeted destruction of harmful HDAC isoenzymes, which may improve treatment accuracy. Monitoring target engagement, optimizing dose selection, and facilitating patient stratification will all depend on the integration of trustworthy epigenetic biomarkers, such as transcriptional signatures, HDAC activity assays, and histone acetylation profiles. When taken as a whole, these developments should help create more individualized and potent epigenetic treatments for neurodegenerative illnesses.

## 6. Conclusions

The complications observed in central nervous system (CNS) diseases continue to fulfill the quest for more specific and efficacious treatments. Epigenetic regulators such as histone deacetylases have become potential targets for intervention, due to which various molecular disease pathologies of these conditions become better understood. This review provides a comprehensive analysis of the role of histone deacetylases (HDACs) in the pathophysiology of major neurological disorders, including Alzheimer’s disease, Parkinson’s disease, Huntington’s disease, and glioblastoma. Part of the problem is the extensive sequence and structural homology between HDAC isoenzymes, which makes it challenging to develop an inhibitor that can specifically modulate a single isoenzyme. The collective evidence discussed highlights that dysregulated HDAC activity contributes to neurodegeneration through multiple converging mechanisms, including transcriptional repression of neuroprotective genes, impaired synaptic plasticity, mitochondrial dysfunction, oxidative stress, defective protein clearance, and sustained neuroinflammatory responses.

A key conclusion of this review is that individual HDAC isoenzymes exert distinct and disease-relevant functions in the central nervous system. Class I HDACs (HDAC1, HDAC2, and HDAC3) primarily drive transcriptional repression and synaptic dysfunction, whereas class II enzymes, particularly HDAC4 and HDAC6, regulate cytoskeletal dynamics, protein homeostasis, autophagy, and neuroinflammatory signaling. Among these, HDAC6 stands out as an especially attractive therapeutic target due to its cytoplasmic localization, role in α-tubulin deacetylation, and ability to modulate protein aggregation and inflammation with minimal impact on global gene transcription.

In parallel, s-triazine (1,3,5-triazine) has emerged as a privileged scaffold for CNS-oriented HDAC inhibitor design, owing to its planar, electron-deficient aromatic core and high functionalization potential. The integration of structure-based design, in silico ADME prediction, and computational modeling has further enabled the development of BBB-permeable and isoenzyme-selective candidates. s-triazine-based scaffolds, known for their chemical stability, tunable functionality, and proven utility in HDAC inhibition, represent valuable structural frameworks for developing next-generation, CNS-directed epigenetic modulators.

Passing the BBB efficiently is still an important standard for CNS-active drugs. A major finding of this review is the growing therapeutic relevance of multi-target-directed ligands (MTDLs). Compounds combining HDAC inhibition with modulation of complementary CNS targets, including phosphodiesterases, NMDA receptors, acetylcholinesterase, MAO-B, and amyloid-β aggregation, demonstrate superior efficacy in cellular and animal models by simultaneously addressing epigenetic dysregulation. These findings support a shift away from single-target strategies toward rational polypharmacology for complex neurological diseases. Except for a few, most HDAC inhibitors have polar functionalities that interfere with passive diffusion into brain tissue. To overcome this, more recent design approaches aim to modulate molecular properties, including minimizing hydrogen bond donors, enhancing lipid solubility, and incorporating structural rigidity to enhance CNS penetration. Advances in zinc-binding groups (ZBGs) that are critical for HDAC binding are also assisting in achieving a more optimal balance between potency and permeability.

Future CNS drug development will increasingly adopt integrated, systems-level strategies that reflect the multifactorial nature of neurological disorders. In order to achieve disease modification rather than symptomatic control, the focus will shift toward logically constructed, BBB-permeant multi-target therapies that combine epigenetic modulation with anti-inflammatory and antioxidant mechanisms. Advances in AI-assisted drug design and structure–activity optimization will accelerate the development of selective HDAC inhibitors and s-triazine-based hybrid molecules with improved efficacy and safety. In parallel, innovative delivery technologies will play a crucial role in enhancing brain targeting and reducing off-target effects. In combination with new delivery technologies and synergistic targets, this holistic approach promises to revolutionize the treatment of challenging CNS disorders and unlock more effective, patient-tailored therapies.

## Figures and Tables

**Table 1:** Integrated PET and AFM features and their applicability in major neurodegenerative disorders.

Imaging Modality	Molecular/Functional Feature	Neurological Disorder(s)	Pathological Target	Key Applicability/Clinical Relevance
PET	Protein aggregation	Alzheimer’s disease, dementia	Amyloid-β, Tau	Early diagnosis, in vivo detection of pathological burden, disease staging
PET	Metabolic dysfunction & neuroinflammation	Alzheimer’s disease, multiple sclerosis, epilepsy	Glucose metabolism, microglial activation	Understanding disease mechanisms, monitoring disease progression
PET	Neurotransmitter dysfunction	Parkinson’s disease	Dopaminergic system	Differential diagnosis, disease monitoring, treatment response
AFM	Nano-imaging & nanomechanics	Alzheimer’s disease	Amyloid-β, Tau	Identification of toxic oligomers, aggregation polymorphism, nanoscale biomarkers
AFM	High-resolution imaging & force spectroscopy	Parkinson’s disease	α-Synuclein	Structure–toxicity correlation, membrane interaction and aggregation pathways
AFM	Morphological, mechanical & chemical analysis (AFM-IR)	Huntington’s disease	Mutant huntingtin (polyQ-expanded)	PolyQ-length-dependent aggregation, structure–toxicity relationship

**Table 2:** Comparison of pan-HDAC and isoenzyme-selective HDAC inhibitors in neurodegenerative diseases.

Feature	Pan-HDAC Inhibitors	Isoenzyme-Selective HDAC Inhibitors	References
Target profile	Broad inhibition of multiple HDAC isoenzymes (Class I, II, and IV)	Selective inhibition of specific HDAC isoenzymes (e.g., HDAC2, HDAC3, HDAC6)	[64,65]
Mechanism of action	Global increase in histone and non-histone protein acetylation	Targeted modulation of disease-relevant epigenetic and non-epigenetic pathways	[64,65]
Therapeutic efficacy	Effective in preclinical models, but often lacks disease specificity	Enhanced efficacy by selectively targeting pathogenic HDAC isoenzymes	[64,65]
Toxicity and side effects	Higher risk of dose-limiting toxicities due to off-target effects	Reduced systemic and neurological toxicity	[64,65]
Blood–brain barrier penetration	Frequently limited or suboptimal	Improved BBB penetration through rational molecular design	[64,65]
Impact on neuronal function	May disrupt normal gene regulation and neuronal homeostasis	Preserves physiological HDAC functions while correcting pathological signaling	[64,65]
Clinical translation	Limited success in neurodegenerative clinical trials	Greater translational potential and ongoing optimization	[64,65]

**Table 3:** Functional roles of histone deacetylase (HDAC) classes and subtypes in nervous system development and regulation.

HDAC Class	Subtype	Roles in Nervous System	Effects/Findings	Disease Association	Key References
Class I	HDAC1	Regulates neurogenesis and gliogenesis; Redundant with HDAC2	Deficiency (usually with HDAC2) causes glial dysfunction and impaired myelination by disrupting the NF-κB → Sox10 → Mpz regulatory axis.	Alzheimer’s disease, Huntington’s disease	[66,67]
	HDAC2	Controls neural progenitor differentiation; Redundant with HDAC1	HDAC2 shows lower expression in mature glial cells and functions earlier in neural development compared to HDAC1.	Alzheimer’s disease, Huntington’s disease	[66,67]
	HDAC3	Essential for brain development	Deletion disrupts cortical and cerebellar organization	Alzheimer’s disease, Huntington’s disease	[66,67]
	HDAC8	HDAC8 regulates neural development, inflammation, chromatin structure, and neurogenesis	Deficiency impairs neurogenesis in neural-crest-derived neuronal lineages.	Alzheimer’s disease, Parkinson’s disease	[68,69]
Class IIa	HDAC4	Negative regulator of neurodevelopment	Inhibits MEF2/CREB transcription; induces neuronal apoptosis	Alzheimer’s disease, Huntington’s disease	[70]
	HDAC5	Promotes neuronal differentiation post-nuclear export, plays a role in the consolidation of contextual and tone dependent fear memories	Triggered via Ca^2+^-CaMK pathway; supports MEF2 activity.	Alzheimer’s disease	[70,71]
	HDAC7	Neuronal survival	Promotes cell survival by repressing c-Jun/AP-1-dependent pro-apoptotic gene expression.	Parkinson’s disease	[72]
	HDAC9	Found in post-mitotic, mature neurons	Linked to neurological regulation, neuronal differentiation, and stress–response pathway.	Alzheimer’s disease, Parkinson’s disease	[73,74]
Class IIb	HDAC6	Regulates dendritic development	Deacetylates α-tubulin; aids microtubule remodeling and dendrite formation	Alzheimer’s disease, Huntington’s disease Parkinson’s disease	[75,76]
Class III	SIRT1	Neuroprotection and regulation of neuronal stress response	Delays neurodegeneration; enhances synaptic plasticity; provides protection in Alzheimer’s and Parkinson’s diseases	Alzheimer’s disease, Huntington’s disease Parkinson’s disease	[28,77,78]
	SIRT2	Myelin maintenance and cell cycle control	Overexpression linked to oxidative stress; regulates oligodendrocyte maturation and myelination.	Parkinson’s disease, Huntington’s disease	[28,77,78]
	SIRT3	Mitochondrial function in neurons	Protects against oxidative damage; supports energy metabolism in the brain	Alzheimer’s disease, Parkinson’s disease	[77,78]
	SIRT4	Less defined in brain	Involved in metabolism; neuro roles still being studied	-	[77,78]
	SIRT5	Linked to antioxidant defense in neurons	Involved in detoxification pathways (e.g., ammonia control); unclear neural role	Parkinson’s disease	[77,78]
	SIRT6	DNA repair and anti-aging in neurons	Supports genomic stability, protects against neurodegeneration. High amount of sirt6 reduces tau stability and promotes its clearance, whereas sirt6 deficiency increases tau stability, elevates hyperphosphorylation, and accelerates tau-mediated neurodegeneration.	Alzheimer’s disease	[77,78]
	SIRT7	Least understood in the brain	Involved in RNA stability and cellular stress response.	-	[77,78]
Class IV	HDAC11	Potential role Oligodendrocyte and neuron maturation	Expression increases during brain maturation.	Alzheimer’s disease, Parkinson’s disease	[79,80]

**Table 5:** Various HDAC inhibitors for Parkinson’s disease therapy.

Compound	Structural Feature(s)	Mechanism of Action	Key Findings	Ref.
Compound **19**	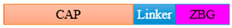	Selective HDAC6 (IC_50_ = 1.8 nM) inhibition; binds Zn^2+^ monodentately	IC_50_ = 1.8 nM (HDAC6); >116-fold selectivity; brain/plasma = 2.1; reduces NLRP3 inflammasome-mediated neuroinflammation.	[119]
	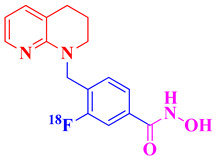			
Compound **20**	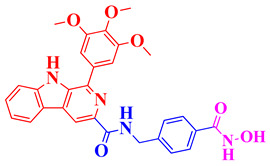	Inhibits HDAC6 (IC_50_ = 28 nM) and HDAC1; Enhances acetylation of mitochondrial NDUFV1 (K28).	Protects SH-SY5Y and dopaminergic neurons; reduces ROS; rescues MPTP mouse model motor deficits; restores TH expression	[120]
Compound **21**	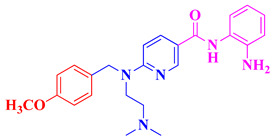	Class I HDAC inhibition (IC_50_ HDAC1 = 1.5 µM); increases histone H3K9 acetylation; transporter-mediated BBB penetration.	PSBBB = 42.4 µL/min/g (3.3× CI-994); sustained epigenetic modulation; proposed strategy for CNS-penetrant HDAC inhibitors	[121]

**Table 6:** Clinical and histological overview of glioma grades preceding glioblastoma.

Grade	Malignancy Level	Tumor Characteristics	Median Survival	Example
I	Non-malignant	Benign, well-differentiated, often curable with surgery	Long-term (often curable)	Pilocytic astrocytoma
II	Relatively non-malignant	Slow-growing, infiltrative, may recur, not aggressive	Varies (years)	Diffuse astrocytoma
III	Low-grade malignancy	Actively growing, more aggressive, requires therapy	Intermediate (months–few years)	Anaplastic astrocytoma
IV	Highly malignant	Rapid progression, necrosis, high mitotic activity	6–12 months	Glioblastoma multiforme

## Data Availability

Data sharing is not applicable to this article, as it is a review article; no new data were created or analyzed.

## Abbreviations

The following abbreviations are used in this manuscript:

Aβ	Amyloid beta
AD	Alzheimer’s Disease
AChE AFM	Acetylcholinesterase Atomic Force Microscopy
AKT	Protein Kinase B
ALS	Amyotrophic Lateral Sclerosis
Arc	Activity-Regulated Cytoskeleton-Associated Protein
BBB	Blood–Brain Barrier
BDNF	Brain-Derived Neurotrophic Factor
Bcl-2	B-cell Lymphoma 2
Bcl-xL	B-cell Lymphoma-extra Large
CaMK	Calcium/Calmodulin-Dependent Protein Kinase
Cap-QuIC	Capillary-Based Quaking-Induced Conversion
CAS	Chemical Abstracts Service
CNS	Central Nervous System
CREB	cAMP Response Element-Binding Protein
DLB	Dementia with Lewy Bodies
DNA	Deoxyribonucleic Acid
EGR1	Early Growth Response Protein 1
GBM	Glioblastoma Multiforme
γH2AX	Phosphorylated Histone H2AX
HAT	Histone Acetyltransferase
HD	Huntington’s Disease
HDAC	Histone Deacetylase
HDACi	Histone Deacetylase Inhibitor
HGC	Histone Deacetylase 6 Inhibitor HGC
HMEC	Human Mammary Epithelial Cells
IC_50_	Half Maximal Inhibitory Concentration
IFN-γ	Interferon Gamma
IL	Interleukin
JAK/STAT	Janus Kinase/Signal Transducer and Activator of Transcription
LRRK2	Leucine-Rich Repeat Kinase 2
MAPK	Mitogen-Activated Protein Kinase
MEF2	Myocyte Enhancer Factor 2
MRI	Magnetic Resonance Imaging
MTDL	Multi-Target-Directed Ligand
NDUFV1	NADH Dehydrogenase [Ubiquinone] Flavoprotein 1
NEP	Neprilysin
NF-κB	Nuclear Factor Kappa B
NMDAR	N-Methyl-D-Aspartate Receptor
PD	Parkinson’s Disease
PD-L1	Programmed Death-Ligand 1
PDE PET	Phosphodiesterase Positron Emission Tomography
PI3K	Phosphoinositide 3-Kinase
PROTAC	Proteolysis-Targeting Chimera
PSD95	Postsynaptic Density Protein 95
PYK2	Proline-Rich Tyrosine Kinase 2
SAR	Structure–Activity Relationship
SIRT	Sirtuin
STAT3	Signal Transducer and Activator of Transcription 3
TGI	Tumor Growth Inhibition
WHO	World Health Organization
